# EU-27 ecological footprint was primarily driven by food consumption and exceeded regional biocapacity from 2004 to 2014

**DOI:** 10.1038/s43016-023-00843-5

**Published:** 2023-09-14

**Authors:** Alessandro Galli, Marta Antonelli, Leopold Wambersie, Anna Bach-Faig, Fabio Bartolini, Dario Caro, Katsunori Iha, David Lin, Maria Serena Mancini, Roberta Sonnino, Davy Vanham, Mathis Wackernagel

**Affiliations:** 1 Global Footprint Network, Geneva, Switzerland; 2 Impacts on Agriculture, Forests and Ecosystem Services (IAFES), Fondazione Centro Euro-Mediterraneo sui Cambiamenti Climatici (CMCC), Viterbo, Italy; 3 Global Footprint Network, Oakland, CA USA; 4 École de technologie supérieure, Montréal, Québec Canada; 5 Food Lab Research Group (2021 SGR 01357), Faculty of Health Sciences, Open University of Catalonia (UOC), Barcelona, Spain; 6 Department of Chemical, Pharmaceutical and Agricultural Sciences, University of Ferrara, Ferrara, Italy; 7 Department of Environmental Science, Aarhus University, Roskilde, Denmark; 8 Joint Research Centre of the European Commission, Seville, Spain; 9 Centre for Environment and Sustainability, University of Surrey, Guildford, UK; 10 Independent Researcher, Ispra, Italy

**Keywords:** Environmental impact, Agriculture, Sustainability

## Abstract

The European Union (EU) plans to decarbonize the region by 2050. As highlighted by the Green Deal and Farm to Fork Strategy, food systems are essential for this transition. Here we investigate the resource dependence and carbon emissions of the EU-27’s food systems from 2004 to 2014 via an ecological footprint (EF)-extended multi-regional input–output approach, accounting for demand and supply (including trade), and considering multiple externalities. Food contributes towards almost a third of the region’s EF, and appropriates over half of its biocapacity. Average reliance on biocapacity within national borders decreased, while reliance on intra-EU biocapacity increased; yet a quarter of the biocapacity for food consumption originates from non-EU countries. Despite a reduction in both total EF and food EF over the study period, EU-27 residents demand more from nature than the region’s ecosystems can regenerate—highlighting the need for new or strengthened food and trade policies to enable a transformation to sustainable EU food systems.

## Abstract (web summary)

An ecological footprint approach demonstrates that EU-27 residents demand more from nature than the region’s ecosystems can regenerate, with a quarter of biocapacity for food originating outside of the region.

## Main

In the context of persistent global ecological overshoot (https://www.overshootday.org/), the transformation of food systems is one of the biggest challenges of our time^1^, being debated from a variety of perspectives^2,3^. The food system, from farm to fork to disposal, generates substantial pressures and impacts on the environment and contributes to the increasing competition over land and water resources, causing anthropogenic greenhouse gas (GHG) emissions and the loss of biodiversity^4,5^. Food-system emissions already amount to the equivalent of 18 Gt CO_2_ per year globally (in 2015), accounting for something between 34% (ref. ^6^) to 37% (ref. ^7^) of total GHG emissions. Food consumption is a substantial driver of the transgression of planetary limits^4,5,8^, and of large shares of countries’ ecological footprints (EFs)^9^.

By 2050, food production would need to increase by 70% relative to 2009 to meet the food demands of a growing and increasingly urbanized population that is demanding more resource-intensive diets^10^. Recent estimates suggest that total food consumption will increase by 51% relative to 2010 (ref. ^11^); however, yield gains are unlikely to occur without increasing environmental burdens, even when factoring in improvements in efficiency^12^. Over the same period, in a business-as-usual scenario, food-related GHG emissions are expected to grow by 87%, cropland use by 67%, blue water use by 65%, and phosphorus and nitrogen application by 54% and 51%, respectively; this would put key ecosystem processes at risk^5^. A transition to healthier and more sustainable food systems thus requires a global shift in dietary patterns and reductions of food loss and waste, alongside radical improvements in agriculture and food production practices^13^. It will also require the preservation of both domestic and global natural capital to maintain and improve the resilience of food systems in the upcoming decades^14^.

In the European Union (EU), food systems are at a crossroads. The European Commission has recently launched the Farm to Fork Strategy, which is at the core of the Green Deal’s ambitious target to decarbonize the continent by 2050. The Strategy^15^ calls for a broad food-system transformation, with an upcoming legislative framework by 2023. This policy could align EU food systems with the Sustainable Development Goals and the Paris Agreement, despite claims by a few impact assessments that the implementation of the Farm to Fork Strategy could lead to leakage effects in terms of GHG emissions^16^, a decrease in agricultural production^17^, as well as price increases and income losses for producers^18^. As food patterns in the EU are already close to exceed the reference value of 2.49 kg CO_2eq_ per capita per day—used to assess the climate compatibility of diets^19^—supply-side changes alone are probably insufficient to make EU food systems sustainable. Previous research^20–22^ has already shown that shifting to healthy diets could substantially reduce the carbon, water and land footprints of the EU, while also limiting the social risks associated with food production and consumption^23^. Food systems in the EU are also at stake in the framework of the EU Strategic Autonomy, which has gained new momentum with coronavirus disease 2019 (COVID-19) and the conflict in Ukraine, and calls for a more diversified food system, trade policies aligned with EU food sustainability standards and competitiveness, and the fostering of local and regional productions with reduced environmental footprints.

In this Article, against this background, we examine the biomass resources and carbon emissions dimensions of food systems in the EU-27 region and its member countries (and place it in the context of their wider socio-economic development) through a customized version of EF accounting (EFA)^24^—namely an EF-extended multi-regional input–output (EF-MRIO) approach^9^. A country or region’s EF indicates the appropriation of biological resources and ecosystem services^25^ to support the consumption patterns—including food consumption—of that country or region’s residents. This method expresses human demand in terms of equivalent land units or hectare-equivalents—namely global hectares (gha), where each gha represents the annual capacity of a hectare of land (for example, crops, pastures, forests and fishing grounds) with world-average productivity to provide ecosystem services that are useful to people. Although not exempt from limitations (Methods), the use of MRIO to perform the EF analysis enables a detailed assessment of the food consumption and sourcing profiles of EU-27 countries, including EF embedded throughout whole supply chains. By including international trade, this methodology captures the externalization of pressures from EU consumption of final food products to other countries, both intra- and extra-EU, highlighting food sourcing dependencies. While previous studies have investigated food-related EFs at the national and city level in a specific year (for example, see research on Portugal^26^ and the Mediterranean region^9^), or have uniquely analysed the carbon, water or land footprints of a few European countries^27,28^, no analysis has yet comprehensively explored the EU-27 region and all its Member States, simultaneously looking at land appropriation and carbon emissions over a 10 year timespan. In this respect, this paper informs the EU Strategic Autonomy ambition to reduce economic dependence on foreign supply chains in virtually all EU policy areas, shedding light on progresses in the food sector (also in the context of the Farm to Fork Strategy), while responding to emerging calls for the generation and integration of knowledge that supports policy decisions and progress tracking^29^.

## Results and discussion

### The ecological and food footprints of the EU-27 region

**Fig. 1:**
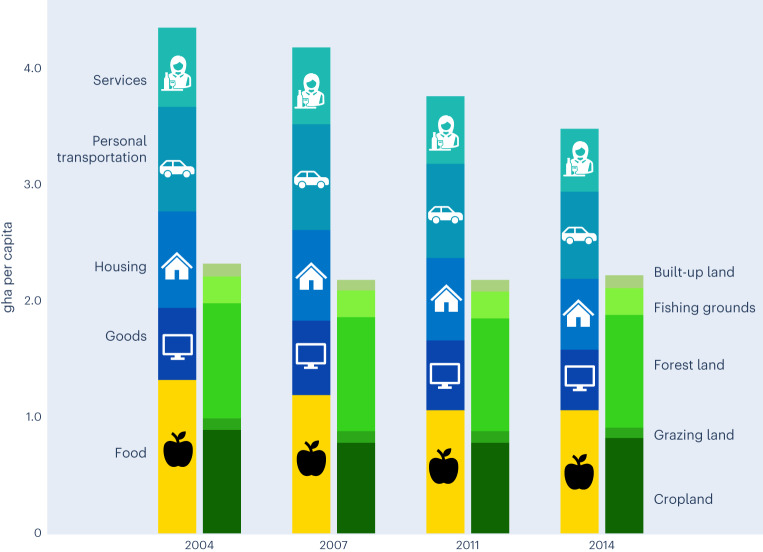
EU-27 EF by consumption categories (left) and BC by land types (right) in selected years (2004–2014). The EF consists of five major categories: food, goods, housing, personal transportation and services. Food, in turn, includes a number of food typologies, that is, bread and cereals; milk, cheese and eggs; fruit; meat; plant-based oils and fats; vegetables; non-alcoholic beverages; fish and seafood; animal-based oils and fats; sugar, jam, honey, chocolate, confectionery and alcoholic beverages; food products not elsewhere classified (n.e.c.). This classification is based on the United Nations COICOP coding system. BC consists of five land types: cropland (for the provision of plant-based food, feeds and fibre products), grazing land (for the production of animal products); fishing grounds (for the production of fish products); forests (for the production of timber and other forest products, and for CO_2_ sequestration); and built-up surfaces (for the provision of shelter and other urban infrastructures).

Over the timespan considered in this study (2004–2014), EU citizens demanded on average a much higher amount of biocapacity (BC) to sustain their overall consumption patterns than what the region could regenerate, leading to an ecological deficit (Fig. 1). Average per capita EF decreased by 20%—from 4.34 gha per capita in 2004 to 3.47 gha per capita in 2014, with reductions observed in all the constituent land types of the EF (Supplementary Fig. A). Despite such decreases, the EU-27 EF was still higher than the per capita BC available within the region, which in turn fell by 4%—from 2.31 gha per capita in 2004 to 2.21 gha per capita in 2014 (https://data.footprintnetwork.org).

Food is a key driver of the EF over the timespan considered. Specifically, we refer to the EF of household food consumption (that is, the BC demanded to provide households with the food they consume) as their food footprint (FF)^9^. At the EU-27 level, FF represents between 28% and 31% of the total EF, appropriating over half of the region’s BC. Food consumption is by far the single largest component in all years considered, followed by personal transportation (21–22%), housing (18–19%), goods, and services (14–16% each). On average, FF accounted for 1.32 gha per capita in 2004 and decreased to 1.06 gha per capita in 2014, while still representing about 30% of the total EU-27 EF. In the majority of EU-27 countries, food consumption is the largest component of the EF (Supplementary Fig. B): this is the case for 21 out of 27 countries in 2004 (excluding Austria, Czech Republic, Finland, Ireland, Luxembourg and Slovenia) and in 2014 (excluding Austria, Czech Republic, Finland, Ireland, Luxembourg and Malta).

### FF by country or region

**Fig. 2:**
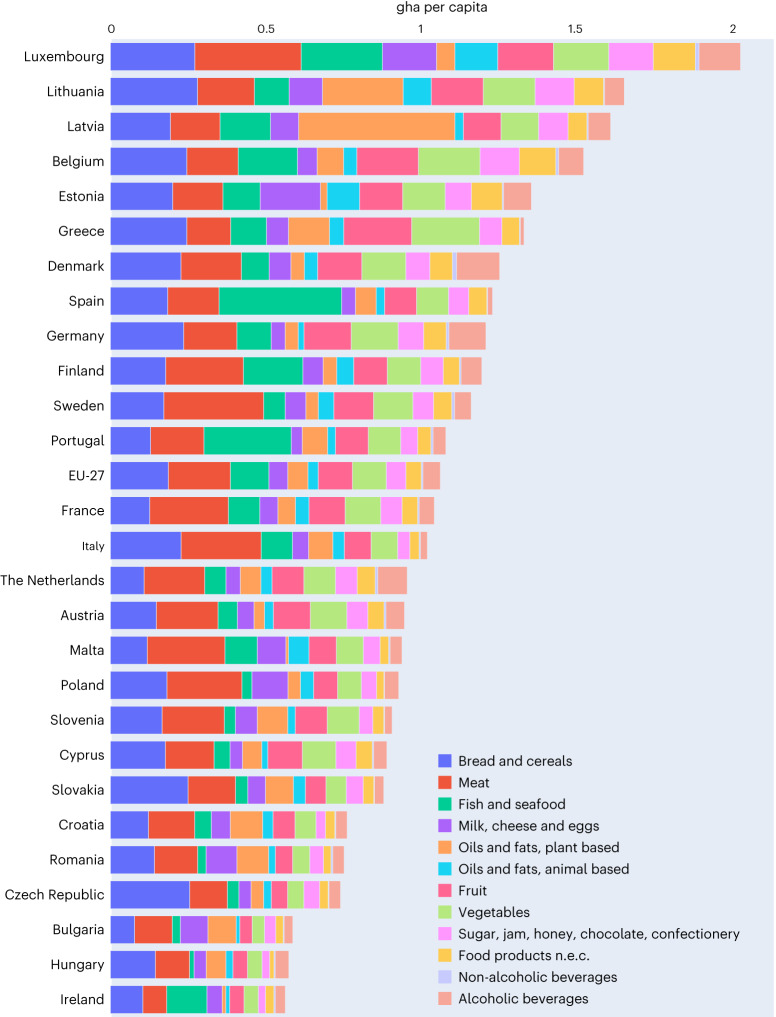
Per capita FF of EU-27 countries, by COICOP macro-categories (2014). The 2004 values are shown in Supplementary Fig. E.

Overall, five countries alone make up nearly 70% of the EU-27 FF: Germany (21%) in Central Europe; France (15%) in Western Europe; Italy (13%) and Spain (12%) in the Mediterranean; and Poland (8%) in Eastern Europe (details are provided in Supplementary Figs. C and D). Each country’s overall FF is determined by its population size and the dietary patterns of its citizens. As shown in Fig. 2, Luxembourg has the highest per capita FF (over 2 gha), followed by Lithuania, Latvia and Belgium (each slightly exceeding 1.50 gha per capita), while—on the other end of the spectrum—Bulgaria, Hungary and Ireland (each with a FF <0.6 gha per capita) have a much lower FF, below that of the EU-27 average (1.06 gha per capita).

Over the timespan considered (2004 values are shown in Supplementary Fig. E), per capita FF values have decreased in most of the countries analysed—especially Cyprus (−36%), Czech Republic (−35%), Hungary, Luxembourg and Romania (−30%)—except for Latvia (+41%), Lithuania (+24%) and Malta (+4%). Nonetheless, FF as a proportion of the total country EF remains quite stable (about 30%) over the period from 2004 to 2014, with a few exceptions—that is, Bulgaria (where it drops from 38% to 31%), Malta (from 28% to 19%), Poland (from 36% to 31%) and Romania (from 43% to 37%). Differences among countries can be explained by different income levels, purchasing powers and lifestyles, including food habits (as discussed below). This is also in line with previous water^30^ and carbon^27^ footprint studies.

**Fig. 3:**
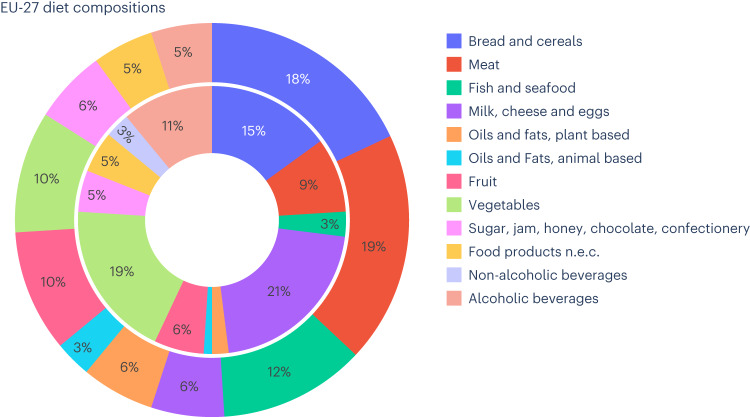
Food supply (inner circle, kg) and FF (outer circle, gha) composition of the EU-27 diet, year 2014. Country-specific pie charts for the year 2014 are shown in Supplementary Fig. F.

Dietary patterns play a key role in determining the FF: on average the consumption of bread and cereals, meat, and fish and seafood collectively contributes to nearly half (49%) of the FF of an EU-27 resident (Fig. 3), although these products constitute just above a quarter (27%) of the approximately 860 kg of food available per person in 2014 (ref. ^31^). Milk, cheese and eggs, and vegetables, contribute 21%, and 19%, of the total food provision in kg, respectively, despite contributing together to just 16% of the FF.

**Fig. 4:**
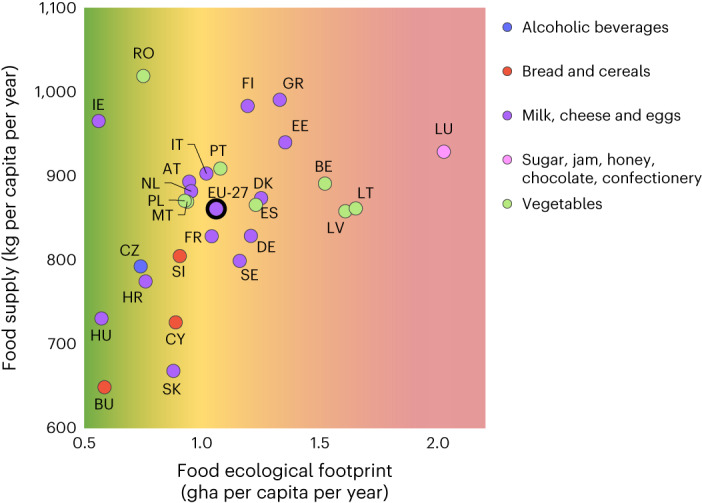
Per capita FF (*x* axis) and food supply (*y* axis) of EU-27 countries in 2014. Dots representing each country on the plot are colour coded according to the COICOP food macro-category its residents consume the most. Colour coding is also used along the *x* axis, depending on whether the per capita FF is <30% (shades of green) or >50% (shades of red) of the EU BC (2.21 gha per capita in 2014), or in between (shades of yellow).

Figure 4 displays the EU countries according to their per capita FF value and food supply in 2014, showing a highly heterogeneous situation: among those with per capita FF values lower than the EU-27 regional average (green-shaded area), we find (1) countries with a lower-than-average food supply whose diets are characterized by a higher intake of bread and cereals (for example, Bulgaria, Cyprus and Slovenia), (2) countries in which milk, cheese and eggs are the predominant sources of animal proteins, irrespective of whether their food supply is lower (for example, Croatia, France, Hungary and Slovakia) or higher (for example, Austria, Ireland, Italy and the Netherlands) than the EU-27 average (Supplementary Fig. F), (3) countries with a higher-than-average food supply whose diets are characterized by a higher intake of vegetables (for example, Malta), and (4) countries whose FF noticeably (~70%) rely on BC from within national borders (for example, Poland and Romania) (Supplementary section 1.3 and Supplementary Fig. G).

**Fig. 5:**
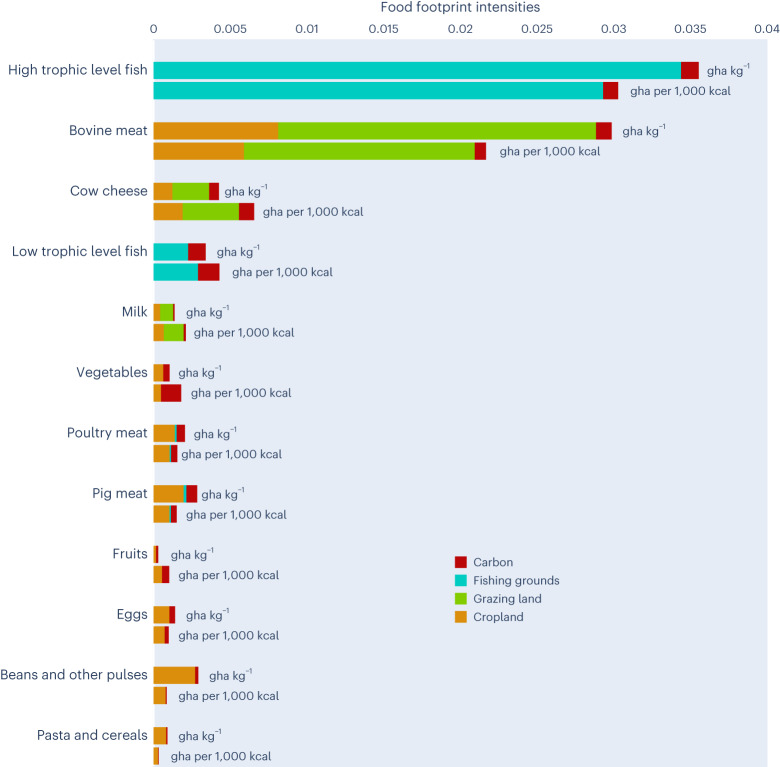
EF intensities of main food item. Ecological footprint intensities, expressed in both gha per kg of product, and gha per 1,000 kcal provided, of selected food items, by land types.

These countries have dietary EF intensities (ranging from 4.2 global m^2^ per 1,000 kcal provided in Ireland to 8.5 global m^2^ in Slovakia) lower than the EU-27 average (8.6 global m^2^ per 1,000 kcal) (Supplementary Table 1), due to a combination of at least four factors: intake (amount and composition), environmental impact of food products, food waste and food sourcing patterns. While this latter is analysed, Fig. 5 shows that the EF intensity (gha per 1,000 kcal) varies by food items and, in line with finding from previous studies^4^, is far larger for animal-based products (for example, meat, fish and seafood) than for plant-based products (for example, vegetables, fruits and pulses).

Consuming meat (especially bovine meat) and fish and seafood (especially high trophic level fishes such us tuna, cod and swordfish) in bigger quantities than the EU-27 average (82 kg and 24 kg per capita, respectively) contributes to increasing a country’s FF. This is the case for Luxembourg (85 kg and 35 kg), Lithuania (81 kg and 33 kg), Portugal (94 kg and 53 kg), and Spain (99 kg and 44 kg) (Supplementary Fig. F). Considering current agricultural and livestock practices, a protein transition away from beef meat could help reduce EU-27 citizens’ FF: cutting the yearly consumption of beef meat (~13 kg per capita according to the Food and Agriculture Organization (FAO)) by half, and replacing the equivalent kcal provision with kcal obtained from poultry or pig meat, or from beans and pulses, would lead to a 6% or 7% reduction, respectively, in the overall per capita FF of a EU-27 resident (from 1.06 to about 0.99 gha per capita).

Food losses and waste also influence the FF of EU-27 countries. Adding together the food waste generated at the retailing, food service and household levels (this being the most relevant component), France generates the largest amount of food waste (135 kg per capita per year)—far above the EU regional average of 113 kg per capita per year, followed by Spain (116 kg), Germany (102 kg), Italy (97 kg) and Poland (94 kg) (calculations based on ref. ^32^). At the current average footprint intensity of these nations’ diets, eliminating food waste could result in a FF reduction between 9% (in Austria and Belgium) and 19% (Malta) and an average reduction of 13% at the EU-27 level (Supplementary Table 1).

### Origins of the EU-27 FF and reliance on trade

**Fig. 6:**
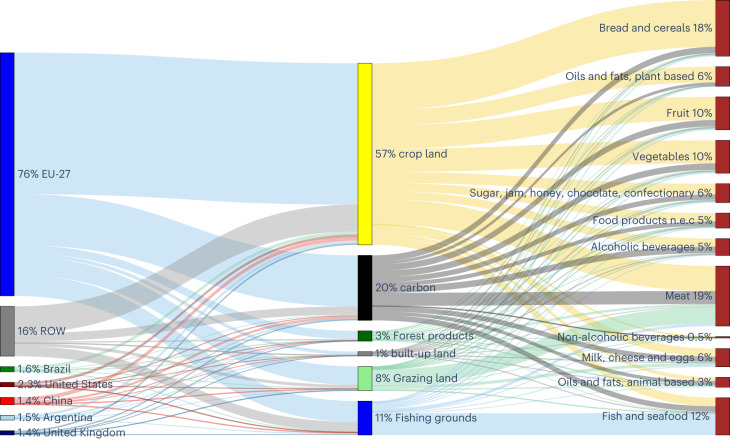
EU-27 FF by food categories, appropriated ecological assets, and origin, in 2014. EU-27 FF broken down by COICOP food macro-categories driving the FF (right column), BC land types (i.e., ecological assets) needed to produce food (middle column), and their geographical origin (left column) in 2014. Year 2004 results are shown in Supplementary Fig. H.

Food consumption in EU countries is found to noticeably depend on food production activities outside national boundaries, as shown in Fig. 6. This figure describes a heterogeneous distribution between food production and food consumption in the EU-27, which is maintained by an intense trading of food and agricultural commodities.

On average, one-fourth of the FF of an EU-27 resident in 2014 relied on BC imports from non-EU countries (24%) (Fig. 6; year 2004 in Supplementary Fig. H). This implies that food consumption within the EU-27 region drives the geographic displacement of ecological asset use throughout the world, although most of such displacement takes place within the EU-27 borders. This figure also shows the ecological assets sustaining human demand for food in the EU-27: in both 2004 and 2014, cropland was by far the main ecological asset sustaining food demand (57% in both years), followed by land for carbon sequestration (21% in 2004 and 20% in 2014), fishing grounds (10% in 2004 and 11% in 2014) and grazing land (8% in both years).

**Fig. 7:**
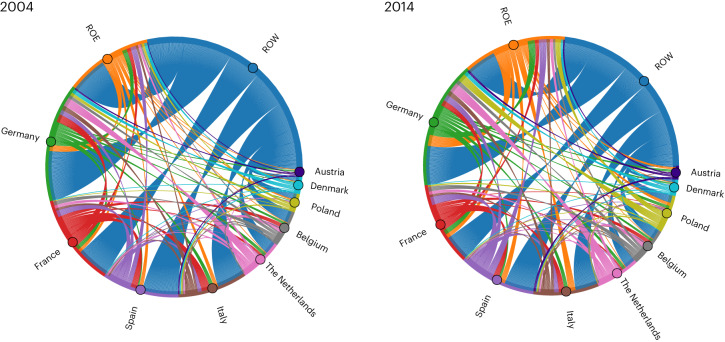
EU-27 food-related biocapacity trade. Inflows and outflows of BC embedded in food trade for the EU-27 region, in year 2004 and 2014. Nine main countries (in terms of their contribution to the total EU-27 FF) are visualized individually, while the others are grouped together as ROE. For the ROW, only outflows of food-related BC are shown.

The share of the EU-27 FF that is reliant on intra-EU BC has slightly increased from 74% in 2004 to 76% in 2014. Although small, this increase is consistent with the increase in intra-regional trade that has occurred over the past few decades as a result of EU trade integration policies (Fig. 7).

Figure 7 displays inflows and outflows of food-related BC for the EU-27 region over the 2004–2014 period, showing that the decrease (from 26% to 24%) in the FF reliance on BC from the rest of the world (ROW) was coupled by an average increase (from 22% to 30%) in the reliance on BC from the rest of Europe (ROE); this is in line with increases in intra-EU trade flows observed by other studies^33^ for virtual water trade (for further country details, see Supplementary Fig. G).

**Fig. 8:**
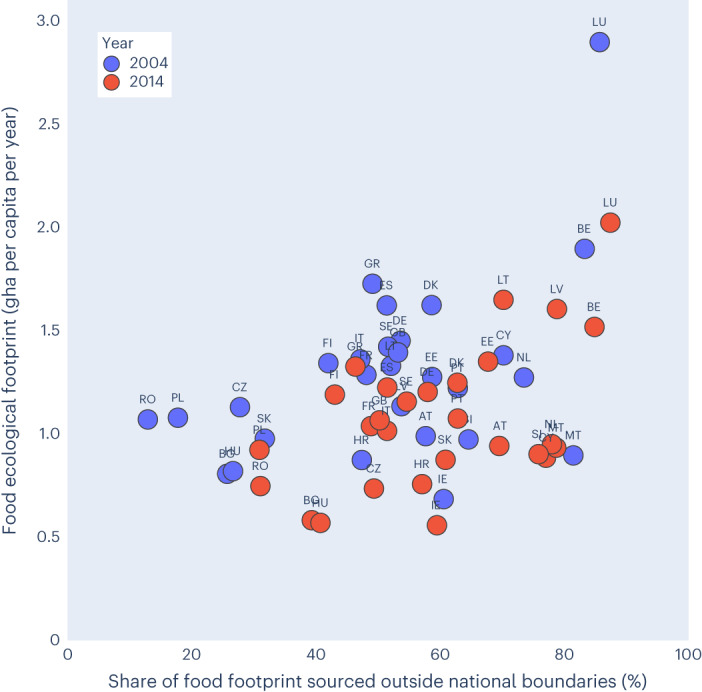
EU-27 FF: countries’ reliance on BC outside national borders in 2004 and 2014. A zoom on the central quadrants (EF between 0.5 and 1.5 gha per person, and external dependency between 40% and 80%) is provided in Supplementary Fig. I.

When plotting per capita FF values against the percentage reliance on BC located outside national borders for the year 2004, EU countries seem to fit within three clusters (Fig. 8). The first is made up of Eastern European countries (Romania, Poland, Czech Republic, Bulgaria, Hungary and Slovakia), which were experiencing a transition phase linked to the accession (recent or upcoming) to the EU, with a share of international FF below 40% and consumption values between 0.75 and 1.2 gha per capita. The second cluster contains the majority of EU countries with 40–80% of FF imports from abroad, and consumption values between 0.75 and 1.75 gha per capita. Countries of very limited size and high population densities (Luxembourg, Belgium and Malta) compose the third cluster, featuring very high levels of BC dependency on other countries (over 80%).

Two main processes can be observed from a longitudinal perspective. First, there is an overall reduction in per capita FFs, with different magnitudes in the EU: larger changes in the central and northern countries (with the exception of increasing FF trends in Estonia, Latvia and Lithuania) and less substantial changes in the Mediterranean region. Second, there is an overall increase in the share of FF sourced outside the national borders. More specifically, in 2004, the average reliance of EU-27 countries on the import of BC from abroad was 48% of the FF, with notable differences between countries: the share of FF sourced from outside national borders ranged from as low as 13–18% (in Romania and Poland), which indicates a high degree of food-related BC sovereignty, to as much as 83–86% in Belgium and Luxembourg, respectively, which signals, in turn, a high degree of dependence on external BC. These differences are largely driven by differences in both consumption patterns and the BC available per person. In 2004, all but five countries (Romania, Poland, Bulgaria, Hungary and the Czech Republic) exceeded 30% reliance on BC from outside their national borders (Supplementary Fig. G). In 2014, the average reliance of EU-27 countries on foreign BC for food increased to 54%: this percentage exceeds two-thirds of the FF per capita in 10 out of 27 countries, including Austria, Belgium, Cyprus, Estonia, Latvia, Lithuania, Luxembourg, Malta, the Netherlands and Slovenia. Interestingly, some of the main exporters of agrifood products show a substantial dependency on BC originating outside national borders. For instance, the Netherlands decreased the proportion of the FF met via domestic BC from 26% to 22% over the period considered, while increasing the proportion sourced from both ROE (from 33% to 36%) and ROW (from 41% to 42%). Recent data (accessible at https://food.footprintnetwork.org/) shows that the Netherlands’ top BC partners include Brazil, as well as Germany and France in the EU. France and Germany, in turn, exhibit a reliance on imports to access food BC that amounted to 49% and 58%, respectively, of their FF in the year 2014 (Fig. 8 and Supplementary Fig. G).

## Science and policy implications of this study

Our study demonstrates a decrease in both the EF and FF of the EU-27 during the decade considered, with a decrease in all the constituent land types of the EF. The reduction in the carbon component of the per capita EF (−21% between 2004 and 2014) can be attributed to a decrease in EU GHG emissions (−31% in 2020 compared with 1990), which is likely to be the result of a general energy decarbonization process in the EU connected with—among other factors—three main policies: (1) the EU Emissions Trading System, (2) the Effort Sharing legislation and (3) the legislation on emissions and removals from land use, land use change and forestry^34^. The decrease in the cropland and grazing land components of the EF (−19% and 34%, respectively), and that in the carbon component, can be explained by the reduction in total meat consumption at the EU level (more specifically, red meat consumption decreased, while consumption of white meat increased) between 2004 and 2014 (ref. ^31^).

During the study period, most EU-27 countries converged towards a reduction in their residents’ per capita FF, coupled with an increased dependency (via trade) on BC (that is, biomass and ecosystem services) from outside their national borders, although primarily from within the EU. This tendency is particularly marked for Eastern European countries, probably as a result of the uptake of EU trade integration policies. Such an evolution is possibly also related to the globalization of food systems, which in turn can be related to the nutrition transition: a shift that is expected to produce a global dietary convergence towards Western-style diets, and associated nutrition-related non-communicable diseases (for example, obesity, type 2 diabetes and hypertension)^35^.

Despite reductions in FF values, our analysis demonstrates that food consumption still represents the largest share of the average per capita EF of EU-27 citizens when compared with the four other categories: goods, housing, personal transportation and services, thus contributing to a growing literature of studies identifying food as one of the largest components of national carbon^27^, water^22,28^ and ecological^26^ footprints in industrialized countries. It also shows the EU-27 is still living beyond a sustainable level, and a significant scope exists for designing, implementing and enforcing policies that facilitate and support food-system transformation.

Through our analysis, we have identified a combination of at least four factors that contribute to the EU-27 FF and that, therefore, require urgent policy intervention. These include: (1) food production (and the environmental impacts associated with it), (2) food intake (amount and composition), (3) food waste and (4) food sourcing patterns. Meanwhile, sustaining a footprint larger than its own territorial BC, as in the case of the EU-27 illustrated here, is possible under three conditions: (1) national natural resources are overused, (2) global commons are used (for example, by emitting excess CO_2_ in the atmosphere beyond domestic sequestration) and (3) the BC embedded in imports is higher than that in exports, thus limiting strategic autonomy and putting resource security at risk^36,37^. By helping understand these factors and conditions and the extent to which they each influence the EU-27 FF, we believe our analysis—although limited by the resolution imposed by a large pan-European assessment—can serve as a starting point to (1) guide remedial actions along the full supply chain of food systems, (2) inform the development of policies and actions at multiple governance levels, both nationally and regionally, and (3) favour multi-sectoral and cross-scalar policies for a more efficient science–policy interface^29^. The type of MRIO-based footprint results provided by analyses such as those in our study, could inform the work being conducted at EU level on modernizing the European agricultural statistics system (EASS) via the EU regulation (2022/2379) on statistics on agricultural input and output adopted within the context of the Strategy for Agricultural Statistics for 2020 and beyond. Insights from this study can also support further research in the field: for instance, starting from our results, dynamic scenarios (at country and regional level) could be developed where high footprint intensity foods are replaced with novel and/or more sustainable foods, or where current food imports are replaced with more sustainable European production.

The EU Green Deal and the Farm to Fork strategies aim to position the EU as a leader of the transition towards more sustainable food systems by improving the sustainability of EU agricultural production; yet, as nearly 25% of the BC needed to support the average food consumption of EU-27 citizens originates from non-EU countries, our analysis suggests that the sole application of Farm to Fork objectives to the domestic agricultural sector is not sufficient to meet the EU decarbonization targets. Rather, it would keep externalizing part of the environmental damages to non-EU countries, as also argued by ref. ^16^. Indeed, with €184 billion in exports and €122 billion in imports in 2020, the EU is among the largest exporters and the third largest importer of agrifood products after the United States and China^38^, with a food system that spans across the whole world. Clearly, the EU-27 shall consider further changes in food sourcing profiles.

We acknowledge that not all EU-27 countries might be endowed with the necessary ecological assets to be able to be self-sufficient and feed their citizens within planetary boundaries (thus avoiding running into an ecological deficit); as such, trade might be key for levelling out potential scarcities in national resources. In practice, however, recent global developments and shocks—such as COVID-19 and the war in Ukraine—have highlighted the vulnerabilities of global supply chains and the risks for the EU-27 region of food trade dependencies. Such considerations have triggered discussion in the EU on how to become less import dependent, which is the main goal behind the widening of the EU Strategic Autonomy to almost all policy areas since 2021. In this respect, our detailed analysis of food trade interdependencies shows an evolution coherent with the EU ambition: EU-27 countries have reduced their dependence on food-related BC from outside the EU (from 26% to 24% of the overall FF) in favour of an increase in intra-EU trade (from 22% to 30%) over the time period considered. Nevertheless, about a quarter of food consumption within the EU-27 still depends on BC located outside the region, which raises the need for new or strengthened food trade policies.

At the same time, our results show that variations in the FF of EU-27 countries are also due to aspects such as food consumption and behaviour trends, for which changes are called for in the Farm to Fork Strategy. This is consistent with previous studies^39,40^, and reinforces the importance of sourcing proteins beyond meat and dairy products, increasing the uptake of plant-rich dietary patterns^41^ and reducing ultra-processed foods^42,43^, as well as portion sizes^44^. In line with previous research^4,45,46^, our analysis demonstrates that animal products (meat, fish, and to a lesser extent, dairy) are highly resource-intensive compared with plant-based foods, by both weight and nutritional unit. Yet, plant-based foods (for example, vegetables) constitute the primary item in the diets of only 8 of the 27 EU countries (Fig. 4 and Supplementary Fig. F). While indicating what the recommended sustainable per capita consumption of certain food items should be is beyond the scope of our study, we found that replacing about half of the beef meat intake with kcal obtained from beans and other pulses could contribute up to a 7% reduction in the per capita FF of EU-27 citizens. The need to increase plant-based protein sources such as pulses and nuts is also called for by recent approaches integrating both nutritional and environmental considerations^13^. At the same time, an additional 13% reduction in FF could be achieved by eliminating food waste. As such, policies aimed at securing sufficient access to sustainable and healthy foods should be promoted to achieve both human and planetary health targets, starting for instance from public procurement and school curricula^44^.

Such changes in food consumption could help address sustainability issues while mitigating the increasing prevalence of non-communicable diseases in EU countries, which are linked to unhealthy diets as modifiable risk factors^47,48^. Developing national food-based dietary guidelines (FBDG) that include both nutritional and sustainability perspectives would be integral to this purpose^44^ as recently found by ref. ^41^.Unfortunately, environmental and socio-cultural aspects have so far been neglected in most FBDG, which have mainly focused on health issues^49^. Fortunately, this historical trend is beginning to be reversed, as is the case in the New Nordic or in the Mediterranean Diet, with countries such as Finland, Germany, Sweden, the Netherlands, Denmark, Spain, Estonia, France and Belgium currently embracing both nutritional and sustainability perspectives in their FBDG^50^.

To conclude, the analysis presented in this paper has two main strengths. Firstly, it explores the EF and, in particular, its food component across the EU-27 region and its Member States, tracing the evolution of land appropriation and carbon emissions over a 10 year timespan. Secondly, the use of an MRIO approach captures the externalization of the impacts of EU consumption to other countries, both intra- and extra-EU, highlighting food sourcing dependencies. The limitations of the study are also acknowledged. The analysis is limited to the decade 2004–2014 due to data availability, and therefore should be updated to better understand current EU-27 food-system challenges, which have also been impacted by the COVID-19 pandemic. Food waste, while embedded in the national per capita FF values, is not clearly singled out in our analysis, making it difficult to assess the reduction in FF that could be achieved through waste prevention as opposed to dietary shifts. Moreover, our study does not adequately assess the possible savings that dietary shifts could generate, which should be the subject of a dedicated dynamic modelling study. Last, but not least, by methodological design, current FF analyses do not track GHG emissions other than CO_2_, thus neglecting some of the environmental pressures associated with the meat sector (for example, the release of methane from bovine meat production^51^, or that of nitrogen and phosphorous from poultry meat production^52^). All these limitations should be addressed by future research.

## Methods

### EF and BC overview

EFA offers a way to measure the resource dimension of the human socio-economic development by comparing the demand humans place on Earth’s ecosystems (that is, biologically productive land and marine areas that provide regeneration) to the amount of ecosystems available^36^. Accounting for such an ecological balance is realized by means of two different metrics, both expressed in gha, of biologically productive hectares with world-average productivity^53^:

- On the demand side, the EF measures the biologically productive land and sea area—the ecological assets—that a population requires to meet all its material demands that compete for ecosystem regeneration. This includes food, fibre, timber, carbon sequestration from fossil fuel burning and space to accommodate built infrastructure.
- On the supply side, BC tracks the areas of ecosystems, adjusted for their respective regeneration rates, available in countries, regions or at the global level.

BC, as tracked by National Footprint and Biocapacity Accounts (https://data.footprintnetwork.org), includes cropland for the provision of plant-based food and fibre products, grazing land and cropland for the production of animal products, fishing grounds (marine and inland) for the production of fish and seafood products, forests for the production of timber and other forest products as well as for climate regulation via CO_2_ sequestration, and built-up surfaces for the provision of shelter and other urban infrastructures.

EFA can be applied at various scales: from a single product to an individual, from a city to a nation, and from a region up to humanity as a whole. Depending on the scale of application, EFA can adopt either a top-down (compound) or bottom-up (component) approach. For a given geographical scale (for example, a city), the compound approach calculates the EF using aggregated national statistics on resource and service flows (for example, data on the total national production, import and export of food, fibres, commodities and so on, thus tracking both direct and indirect flows) and eventually allocating the share of the national total it is responsible for to the level being analysed. Conversely, the component approach calculates the EF by first identifying all the resource and service flows directly and indirectly consumed at that geographical scale (for example, the amount of food, fibres, commodities and the like consumed by the residents of that territory) and then adding-up their individual footprint values. The first approach is most commonly used for assessments at global and national scale, while the latter is preferred in product- or company-level assessments, it being data intensive and prone to truncation errors in tracking indirect flows. Within this study, the top-down approach has been used.

When adopting a consumer-based approach, a country’s EF is calculated by tracking the BC (that is, crop, grazing, forest, fish, built-up and carbon-uptake land) used by national production activities and then adding the ecological assets embedded in imported goods and subtracting those embedded in exported goods. While country-level EF analyses usually rely on physical trade flows data^24^, tracking countries’ food consumption and sourcing profiles requires the traditional footprint method to be extended by means of the Global Trade Analysis Project (GTAP) 10 MRIO model^54^, in what is called an EF-MRIO approach. The analysis presented here covers all 4 years provided by the GTAP 10 model: 2004, 2007, 2011 and 2014. The years investigated in this study thus fully depend on the time horizon available within the GTAP database.

While both the traditional EF approach^24^ and the EF-MRIO approach^9^ refer to the EF of final net consumption activities, differences exist in the way in which consumption values are derived. The traditional approach uses physical statistics on production and trade to derive consumption EF values of the country by first tracking the ecological assets appropriated by national production activities (EF_P_) and then adding the EF embedded in imported goods (EF_I_) and subtracting that embedded in exported goods (EF_E_), as shown in equation (1).

1 $$\mathrm{{EF}}_{{\mathrm{C}}}=\mathrm{{EF}}_{{\mathrm{P}}}+\mathrm{{EF}}_{{\mathrm{I}}}-\mathrm{{EF}}_{{\mathrm{E}}}$$

Irrespective of whether it is locally produced (P), imported (I) or exported (E), the EF of each single product or good *i*, is calculated as in equation (2).

2 $$\mathrm{{EF}}=\frac{{P}_{i}}{{Y}_{{\mathrm{W}},i}}\times \mathrm{{EQF}}_{i}$$

where *P* is the amount of each primary product or good *i* that is harvested (or carbon dioxide emitted) in the nation, *Y*_W,*i*_ is the annual world-average yield for the production of the product or good *i* (or its carbon-uptake capacity in cases where *P* is CO_2_), and EQF_*i*_ is the equivalence factor for the land use type producing products *i* (for the full list of products considered and their data sources, see Supplementary Table 2).

The EF-MRIO approach uses the standard methodology (as in equation (2)) and physical input data to calculate the EF of national production activities (EF_P_) but then derives national EF of consumption (EF_C_) by using monetary trade flows to estimate the footprint embedded in global trade flows, as per equation (3):

3 $$\mathrm{{EF}}_{{\mathrm{C}}}={F\times (I-A)}^{-1}\times {D}_{N}$$

where:

- *F* is the environmental extension matrix (direct EF_P_ of sectors normalized per unit of sector output, which is expressed in gha US$^−1^) derived from the initial allocation of EF_P_ for the six assets/land types (crop, grazing, forest, built-up and carbon-sequestration land, as well fishing grounds) to each of the 65 producing economic sectors identified by GTAP 10 (ref. ^9^);
- *D*_*N*_ is the country total final demand for goods, expressed in US$;
- *I* is the identity matrix;
- *A* is the technical coefficients matrix (representing the Leontief inverse), which reflects the monetary exchange between each sector to produce one currency unit worth of output from a specific sector of the economy.

To run the EF-MRIO model, six environmental extension tables are required, which initially allocate the EF of production (EF_P_) for crop, grazing, forest, built-up and carbon-uptake land, as well fishing grounds, to each of the 65 producing economic sectors identified by GTAP 10. The EF_P_ for cropland is allocated to GTAP sectors 1 to 8, the EF_P_ for grazing land is allocated to GTAP sectors 9–12, the EF_*P*_ for forest land is allocated to sector 13 and that of fishing grounds to sector 14, the EF_P_ for carbon-uptake land is allocated to each sector according to its share of the total emissions as provided by GTAP’s energy-environmental extension, and the EF_P_ of built-up land is assigned to each sector depending on the sector’s value added to the country’s GDP (for the full list of GTAP 10 sectors, see Supplementary Table 3). While a global MRIO model is used to calculate the EF of trade flows among 65 economic sectors of 141 regions of the world, the EF results are then provided in this paper are for the sole use of the EU-27 region and its member countries.

Once the resource requirements of each sector in the economy are calculated, as well as the household final demand for each economic sector—including both food-related and food-unrelated sectors^26^—the household final demand is re-categorized into the United Nations Classification of Individual Consumption According to Purpose (COICOP) consumption categories using a sector-to-household (that is, GTAP-COICOP) concordance^9^. We thus refer to the EF of household’s food consumption (that is, the BC demanded to provide households with the food they consume) in each EU-27 country as the country’s FF or food EF. While the results here are presented as individual consumption categories, they represent the entire EF associated with the final demand for each food consumption category. This includes both the direct and indirect demand by each EU-27 country residents for the cropland (directly to produce food crops and indirectly to produce livestock feed crops), grazing land (to produce meat) and fishing ground (directly to produce fish and seafood products and indirectly to produce livestock feed) footprint components, and their indirect demands for the carbon (from CO_2_ released due to food production/cultivation and trade), forest (wood and fibres used for paper and infrastructure) and built-up (land occupied by food industries) components of the EF. The value for the EF associated with COICOP category 01.1.2 or ‘meat’ consumption, for example, includes supply chain or indirect footprints associated with: (1) all cropland that was used to produce the feed purchased by the livestock industry, (2) all cropland used to produce the fibres purchased by the textile industry (to produce clothing and or natural fibres then purchased by the livestock industry), (3) all forest products purchased by livestock industries to build wood pasture fences, (4) all fishing grounds footprint associated with fish-derived fertilizers eventually used to produce crop-based feed, (5) the built-up land associated with infrastructure such as buildings associated with the supply chain, and (6) the land needed to sequester the CO_2_ emitted by vehicles used to transport livestock.

While EF quantifies human demand, BC acts as an ecological benchmark and quantifies nature’s ability to meet this demand. BC is calculated as in equation (4) and, for each country, it provides an assessment of the regenerative capacity of the country’s ecosystems.

4 $$\mathrm{BC}=\sum _{i}{A}_{N,i}\times \mathrm{{YF}}_{N,i}\times \mathrm{{EQF}}_{i}$$

where:

- *A*_*N*,*i*_ is the bioproductive area that is available for the production of each product *i* at the country level;
- YF_*N*,*i*_ is the country-specific yield factor for the land use type producing products *i*, which compares national yields to global average yields for such land use type; and
- EQF_*i*_ is the equivalence factor for the land use type producing each product *i* compared with average land.

Ecosystems such as croplands, grazing lands and fishing grounds produce both food and non-food biomass such as cotton and other fibres; given the lack of detailed data to clearly trace the end use of the produced biomass, it is not possible to quantify the sole food-related BC available in the EU-27 region and its member countries. Hence, the food EF versus BC eco-balances provided in this study are likely to underestimate the actual pressure that food consumption places on each country’s ecosystems.

### EF intensity of national diets

The footprint intensity of each country’s dietary consumption pattern (that is, its food EF intensity) provides information on the amount of BC needed by the food system of that country to provide residents with food. Footprint intensities are useful for cross-country comparisons and for assessing footprint reduction potentials. Footprint intensities are calculated by dividing the annual per capita FF of a country (expressed in gha per capita per year) by that country’s annual food supply data drawn from the FAO Food Balances (FBS) (expressed in kg per capita per year and kcal per capita per year), and are thus expressed in both gha kg^−1^ and gha kcal^−1^; for ease in data and results visualization, the latter is then converted in global square metres (global m^2^ per 1,000 kcal). FF intensities were here calculated by COICOP food macro-categories (Supplementary Table 1) and a few key food items (Supplementary Fig. G). While calculating footprint intensities for key products is straightforward, their calculation for COICOP food macro-categories requires matching the FBS database with the COICOP categories following the procedure stated here below.

Each FAO’s Food Balance Sheet presents a comprehensive picture of a country’s food supply during a reference period. For each food item—that is, each primary commodity and a number of processed commodities available for human consumption—data on the sources of supply and its utilization are shown. On the utilization side, a distinction is made between multiple food uses, including data on the food supplies available for human consumption. To calculate FF intensities for the EU-27 countries (gha per kg of product), food supply data (expressed in kg per capita per year) was first downloaded from the FBS database^31^ for the years 2014–2018 for each country. Supply quantity data from the FBS is provided per broad categories of food commodities (for example, item #2601 tomatoes and products) and each category thus needs to be associated with the COICOP food macro-categories classification used in the FF calculation.

The FBS-COICOP concordance is straightforward for most categories, although a few FBS categories contain several food derivates that fit into different COICOP categories: for instance, FBS category #2601 tomatoes and products includes tomato, tomato juice, paste of tomatoes and peeled tomatoes, which fit into different COICOP categories (Supplementary Fig. J). For these FBS categories, an intermediate step relying on the Central Product Classification (CPC) version 2.1 (ref. ^55^) is used to divide the FBS category into multiple subcategories. CPC is used as it represents an international coding standard providing a complete product classification covering all goods and services, with a correspondence to both the FAO FBS classification and the UN COICOP classification. Subcategories (mostly derived products) from CPC classification are then matched to COICOP subcategories based on the following allocation method: 2017 world crop trade data (that is, import data) drawn from the FAO^56^ are used to calculate the share of specific subcategories (for example, tomato juice) over the total import of all related items (for example, all tomato items); the obtained allocation percentage is then used to split the FAO food supply data into different COICOP categories (Supplementary Fig. J).

By using such a method, the quantities of derived products that are imported (production side) are assumed to reflect the same proportion of derived products that remains and are consumed in the country (consumption side). Lacking better data, we believe this represents a useful first approximation to be able to allocate FBS data into COICOP food subcategories. Once the concordance between FBS categories and COICOP categories was finalized, including the allocation shares of few items, the final supply data (kg per capita per year) were summed up in the 12 COICOP macro-categories for all EU countries. Finally, FF values (gha per capita per year) were divided by the relative food supply data (expressed in kg per capita per year or kcal per capita per year) for each EU-27 country to derive FF intensities (expressed in gha kg^−1^ and gha kcal^−1^, respectively).

### Ecological waste data

Food waste data are directly extracted from the 2021 United Nations Environment Programme Food Waste Index Report^32^ and refers to the amount of waste produced within each country by (1) households, (2) food services and (3) the retails sector. Country-specific values are drawn directly from the database without any manipulation/adjustment, while population data is used to derive population-weighted food waste averages (at household, service and retail level) for the EU-27 region.

### Strengths and limitations of the analysis

EFA focuses broadly on human metabolism. It aims to measure whether or not humans are able to live within the overall ecological budget of the planet. Answering this research question requires trade-offs between scope and resolution: as EF employs a wide scope and systemic approach to assess the impact of multiple pressures that are usually evaluated independently, it is characterized by a reduced resolution in its capacity to assess impacts on each single land component. The carbon footprint component of the EF, for instance, only tracks the bioproductive area that would be needed to sequester CO_2_ emissions, leaving out other key GHGs (for example, methane), which are substantial when assessing the sustainability of meat production and consumption. Other EF limitations include: (1) it tracks pressure on ecosystems, but does not quantify the immediate consequences of such pressures on ecosystem health, such as soil degradation or overfishing, (2) it measures flows rather than stocks of resources, and (3) it only accounts for ecosystems where solar energy is captured by autotrophic organisms (that is, photosynthesis) to create any form of biomass humans find useful, leaving out many of the regulating, maintaining and cultural services that the planet’s ecosystems provide to humans. The results presented in this study shall thus be considered as an underestimation of the full pressure that food production, consumption and trade activities place on the planet’s ecosystems.

Concerning the integration of the traditional EF methodology with an MRIO model to trace pressures connected with the consumption activities of EU-27 citizens (food, more precisely), we used the MRIO GTAP version 10 model^54^, with data availability for the years 2004, 2007, 2011 and 2014, and with specific product, sector and country (dis)aggregations. Many other MRIO models exist, including EXIOBASE, FABIO and EORA. These models differ in country resolution, sectoral/product (dis)aggregation and time coverage. Some models are provided open-access, others not. Efforts are being done in the MRIO community to increase product and country disaggregation, such as recently for EXIOBASE^57^. As with any modelling effort, the choice of a specific EE-MRIO model with its specifications influences the final results of an environmental footprint assessment. Ideally, multi-model assessments should be conducted, thereby providing a sensitivity analysis. However, such an approach is extremely resource and time consuming. This would require an analysis on its own, which was outside the scope of this paper. Such a multi-model analysis has been recently conducted by ref. ^58^ confirming that consumption-related environmental footprint results for a geographical region can differ substantially, depending on the MRIO model used. The results in our paper are therefore not to be regarded as absolute. However, this does not influence the temporal evolution analysis we show.

### Statistics and reproducibility

This study uses a known environmental accounting methodology (that is, EF) to assess the impacts associated with food systems in the EU-27 region and each individual Member State. No method was used to determine sample size as the footprint assessment was conducted by means of national statistical data drawn from official international databases and referring to all residents. Attempts to repeat the analysis (by different researchers via different data analyses) were successful.

### Inclusion and ethics statement

The research conducted in this study has included local (EU) researchers throughout the research process—study design, study implementation, data ownership, intellectual property and authorship of publications. We thus deem the research reported in this study to be locally relevant.

Roles and responsibilities of authors were agreed among them ahead of the research, but no specific capacity-building plan for local researchers was discussed or put in place.

No approval by an ethics review committee was necessary; the findings of our research do not result in stigmatization, incrimination, discrimination or otherwise personal risk to participants, and the research does not involve health, safety, security or other risk to researchers. No biological materials, cultural artefacts or associated traditional knowledge was transferred out of any country.

Regional (EU) and local (country-level) research relevant to this study has been taken into account in the references cited in the study.

### Reporting summary

Further information on research design is available in the Nature Portfolio Reporting Summary linked to this article.

### Supplementary information

Supplementary Information Supplementary Figs. A–J, Tables 1–3 and additional references.

Reporting Summary

Supplementary Data 1 An excel file with five tabs containing source data.

## Data Availability

Data supporting the findings of this study is available from multiple sources (for example, FAO, IEA and GTAP) as described in Methods (for further details on each individual dataset and how to access it, see Supplementary Table 2). Some of these data are freely available (and provided as raw data in the Excel file ‘EU-27 Food Footprint_source data.xlsx’) while restrictions apply to the availability of other data (that is, GTAP version 10), which was used under licence for the current study, and is thus not publicly available. Data other than those included in the ‘EU-27 Food Footprint_source data.xlsx’ file are available from the corresponding author upon request. Visualization of aggregated results is publicly available at https://food.footprintnetwork.org/.

## Code availability

The mathematical algorithms used to obtain the results of this research are describe in details in Methods. The MATLAB and mySQL codes used to run the analysis, however, are not publicly available.
