# Supplementary material for: EU-27 ecological footprint was primarily driven by food consumption and exceeded regional biocapacity from 2004 to 2014
Source: Nat Food. 2023 Sep 14;4(9):810–22. doi: 10.1038/s43016-023-00843-5 (PMC10513931; doi:10.1038/s43016-023-00843-5)
Supplement: Supplementary file 1 — Supplementary Figs. A–J, Tables 1–3 and additional references. [file 43016_2023_843_MOESM1_ESM.pdf]

nature food

Article

https://doi.org/10.1038/s43016-023-00843-5

EU-27 ecological footprint was primarily driven by food consumption and exceeded regional biocapacity from 2004 to 2014

In the format provided by the authors and unedited

Supplementary Material

Content:

- **Figure A.** EU-27 per capita Ecological Footprint by land types and consumption categories in 2004 and 2014.
- **Figure B.** Ecological Footprint of consumption for EU-27 countries broken down by main consumption categories, in 2004 and 2014.
- **Figure C.** A regional overview of the food-related EF (FF), in the year 2014.
- **Figure D.1-5** EF of Germany, France, Italy, Spain and Poland by main consumption categories (a) and their FF by COICOP food macro-categories and land types (b), in selected years between 2004 and 2014.
- **Figure E.** Per capita Food Footprint of EU-27 countries, by COICOP macro-categories (2004).
- **Figure F.** Food Supply (inner circle, kg) and Food Footprint (outer circle, gha) composition of the dietary patterns of EU-27 countries, year 2014.
- **Figure G.** Food Footprint of EU-27 countries by geographical origin, in year 2004 and 2014.
- **Table 1S.** Dietary Footprint intensities and food waste values for EU-27 countries, year 2014. The potential Food Footprint reduction that could be obtained by reducing food waste to zero while mainitaining current Footprint intensities is provided in the last column.
- **Figure H.** EU-27 Food Footprint (FF) by food macro-categories (right column), appropriated ecological assets (central), and food origin (left) in 2004.
- **Figure I** EU-27 Food Footprint: countries' reliance on biocapacity outside national borders in 2004 and 2014 – focus on central quadrants with EF ranging between 0.5 and 1.5 gha per person, and external dependency ranging between 40% and 80%. Arrows are used to connect country's positions in the two years; for arrows originating from, or going outside the zoomed boxes please refer to Figure 6 in the main text.
- **Table 2S.** Input data for the calculation of the Ecological Footprint of national production.
- **Table 3S.** GTAP 10 sectors' list.
- **Figure J.** FBS macro-categories that were split in sub-categories through the CPC classification to match with proper COICOP categories. The % of allocation of each sub-category is also shown.
- **References (cited in Tables)**
- **References (further readings)**
- **Source Data file:** EU-27 Food Footprint_source data.xlsx

Figure A. EU-27 per capita Ecological Footprint by land types and consumption categories in 2004 and 2014.

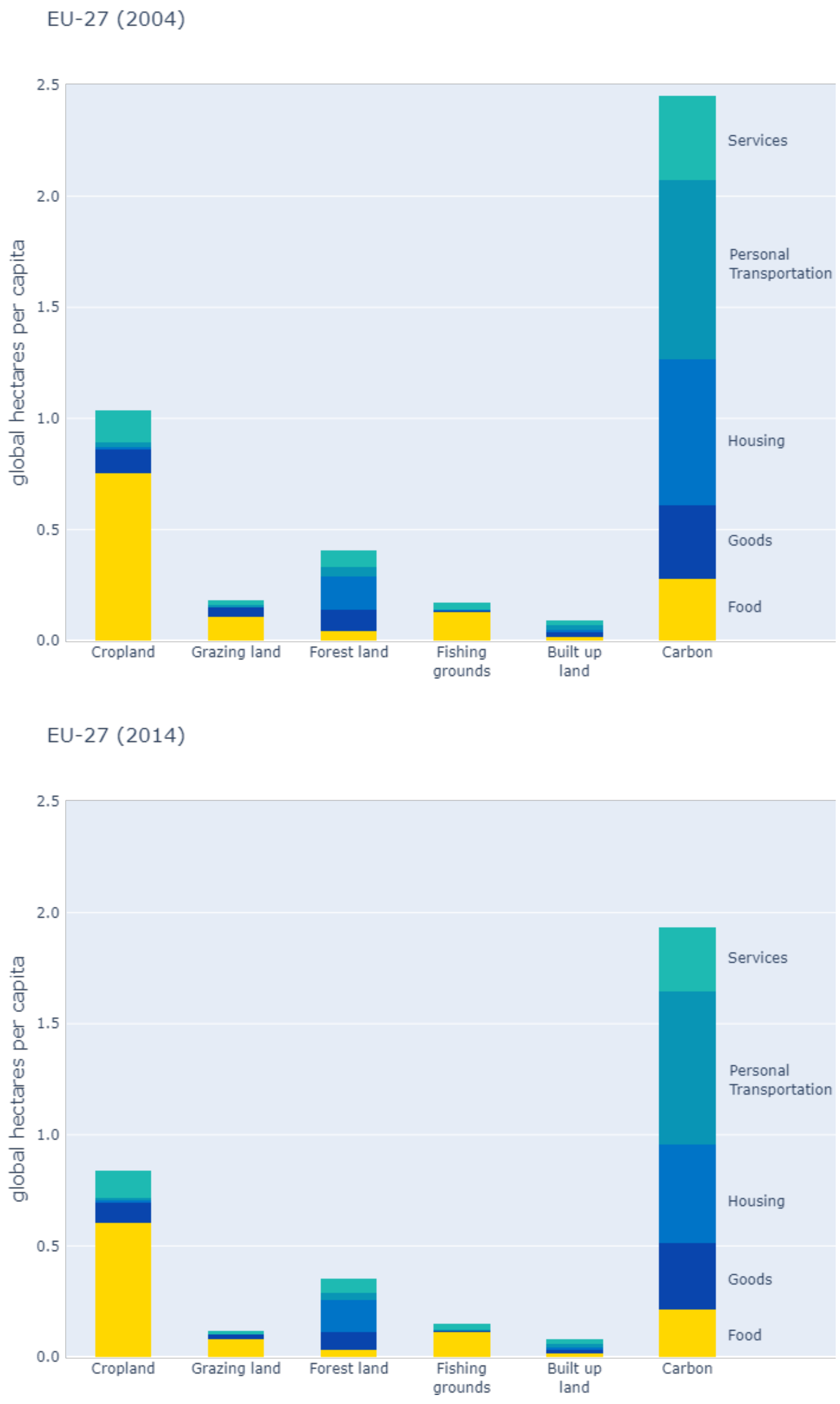

Figure B. Ecological Footprint of consumption for EU-27 countries broken down by main consumption categories, in 2004 and 2014.

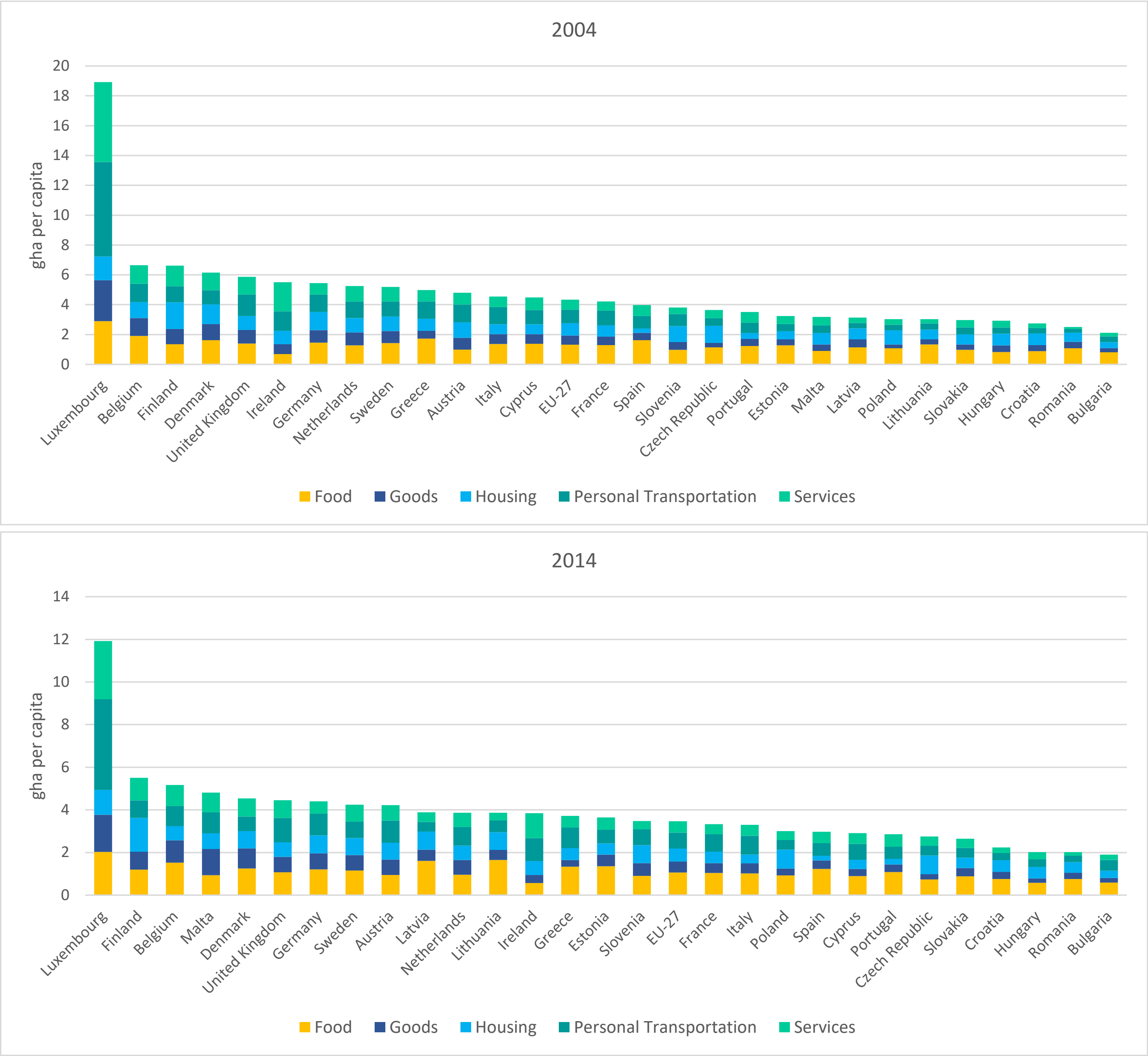

Figure C. A regional overview of the food-related EF (FF), in the year 2014.

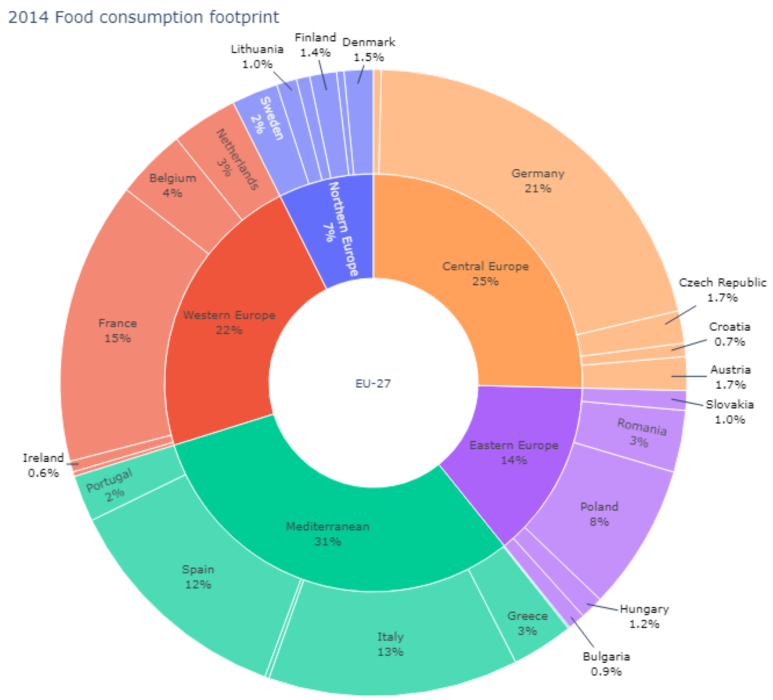

Figure D.1 EF of Germany by main consumption categories (a) and its FF by COICOP food macro-categories and land types (b), in selected years between 2004 and 2014.

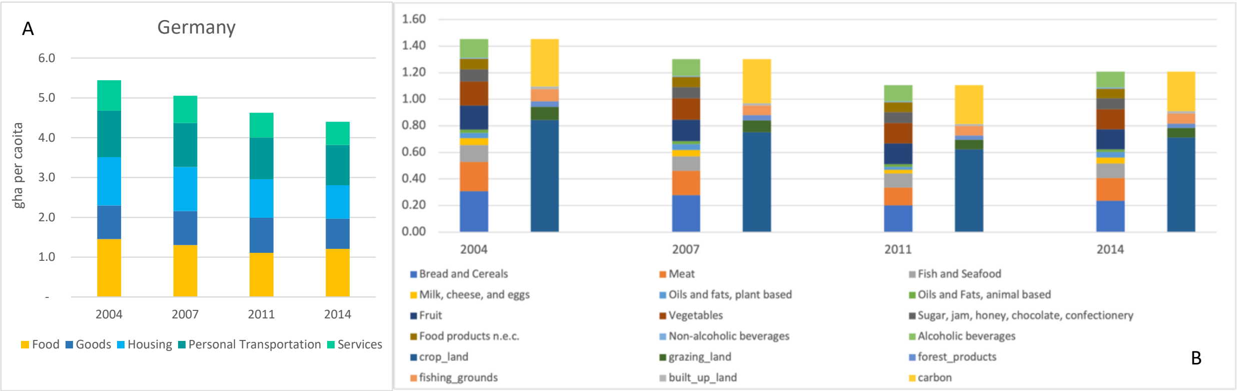

Figure D.2 EF of France by main consumption categories (a) and its FF by COICOP food macro-categories and land types (b), in selected years between 2004 and 2014.

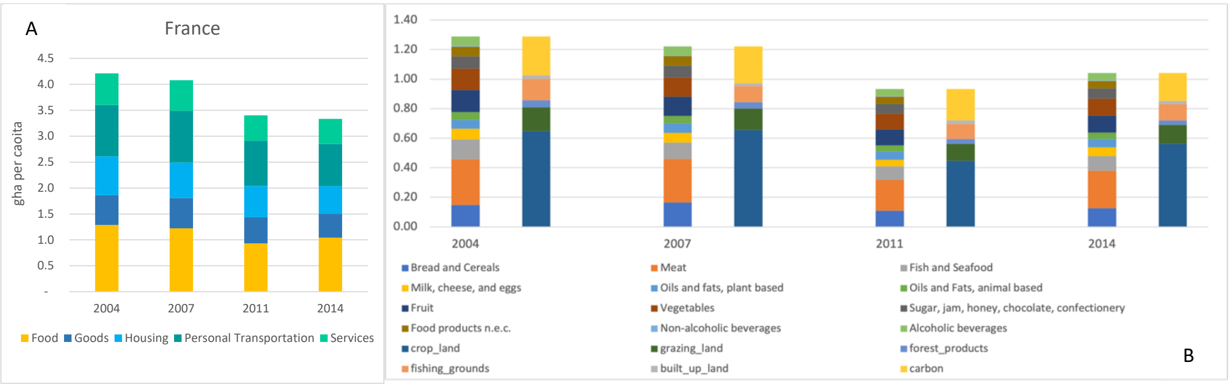

Figure D.3 EF of Italy by main consumption categories (a) and its FF by COICOP food macro-categories and land types (b), in selected years between 2004 and 2014.

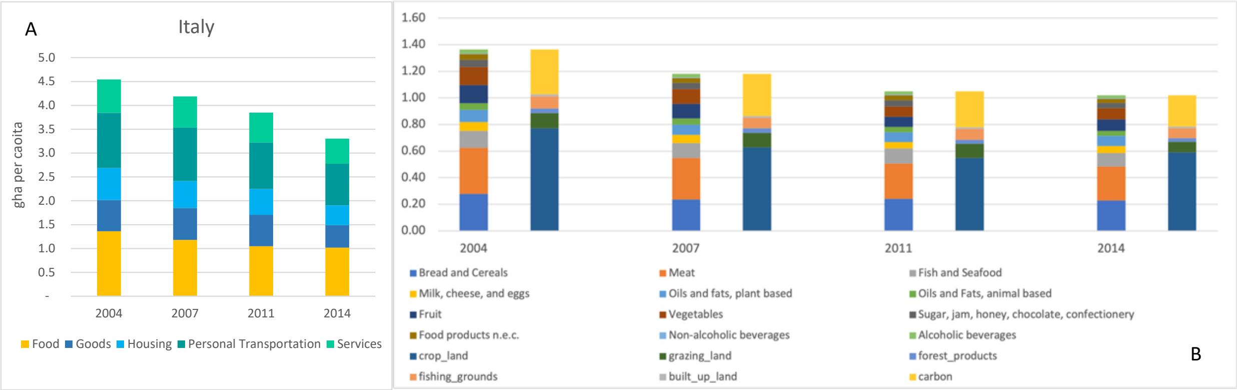

Figure D.4 EF of Spain by main consumption categories (a) and its FF by COICOP food macro-categories and land types (b), in selected years between 2004 and 2014.

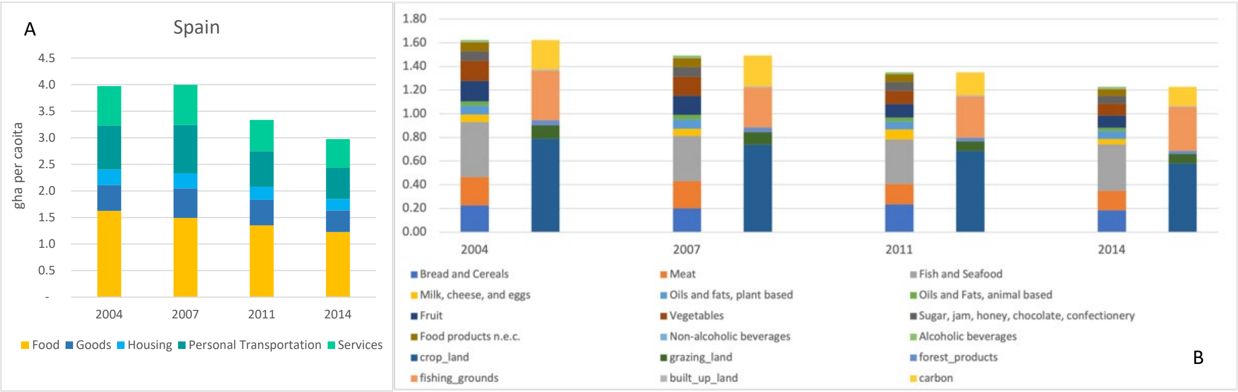

Figure D.5 EF of Poland by main consumption categories (a) and its FF by COICOP food macro-categories and land types (b), in selected years between 2004 and 2014.

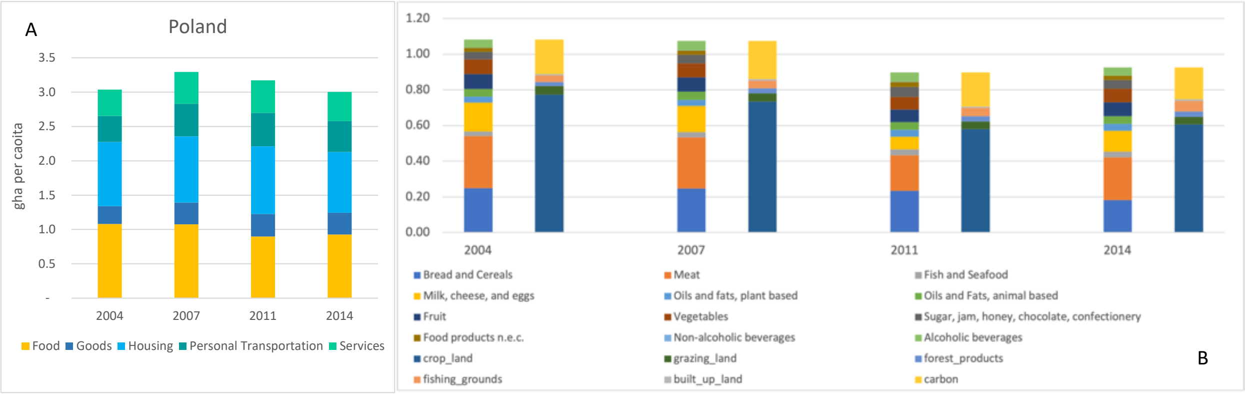

Figure E. Per capita Food Footprint of EU-27 countries, by COICOP macro-categories (2004).

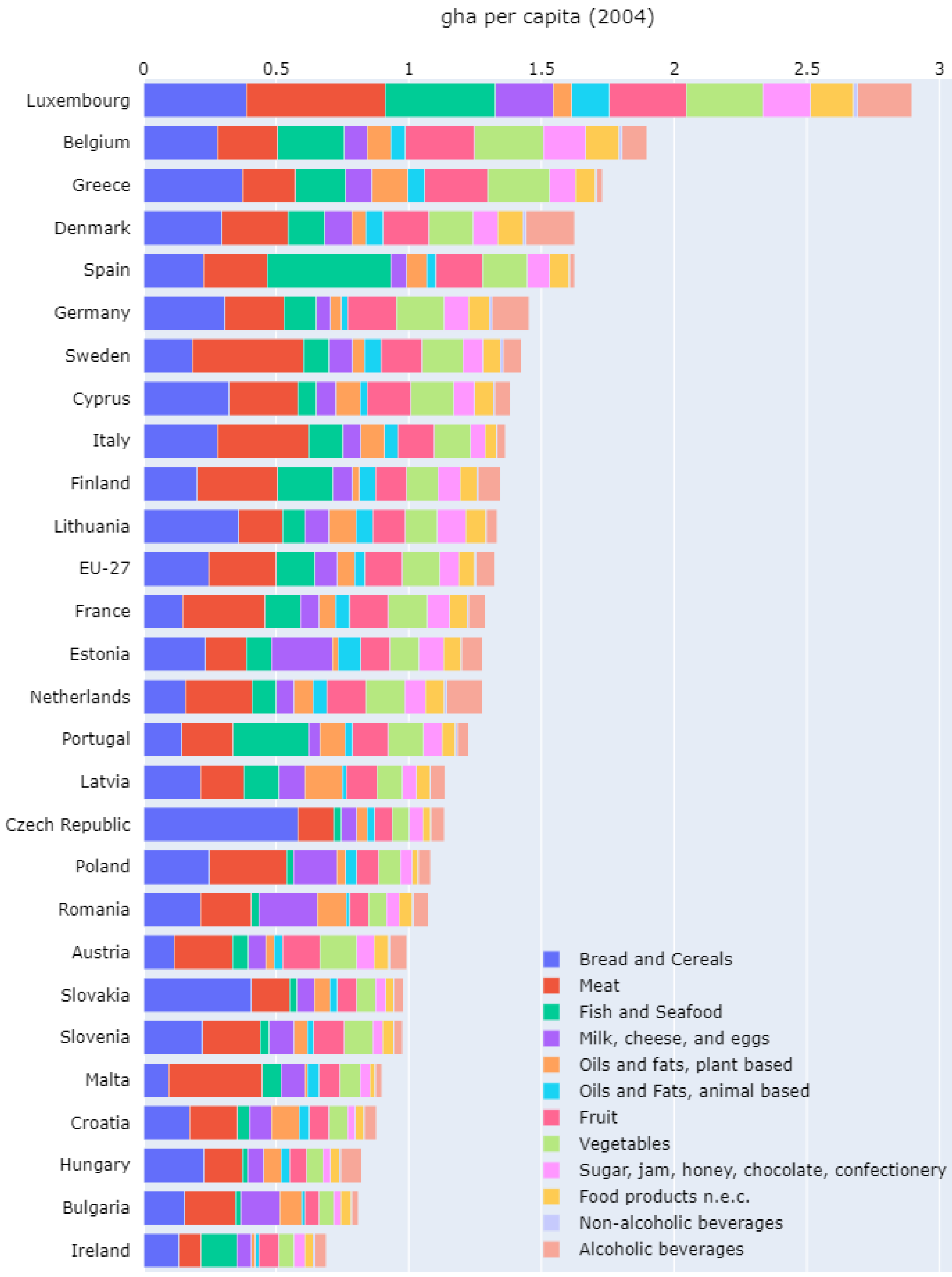

Figure F. Food Supply (inner circle, kg) and Food Footprint (outer circle, gha) composition of the dietary patterns of EU-27 countries, year 2014.

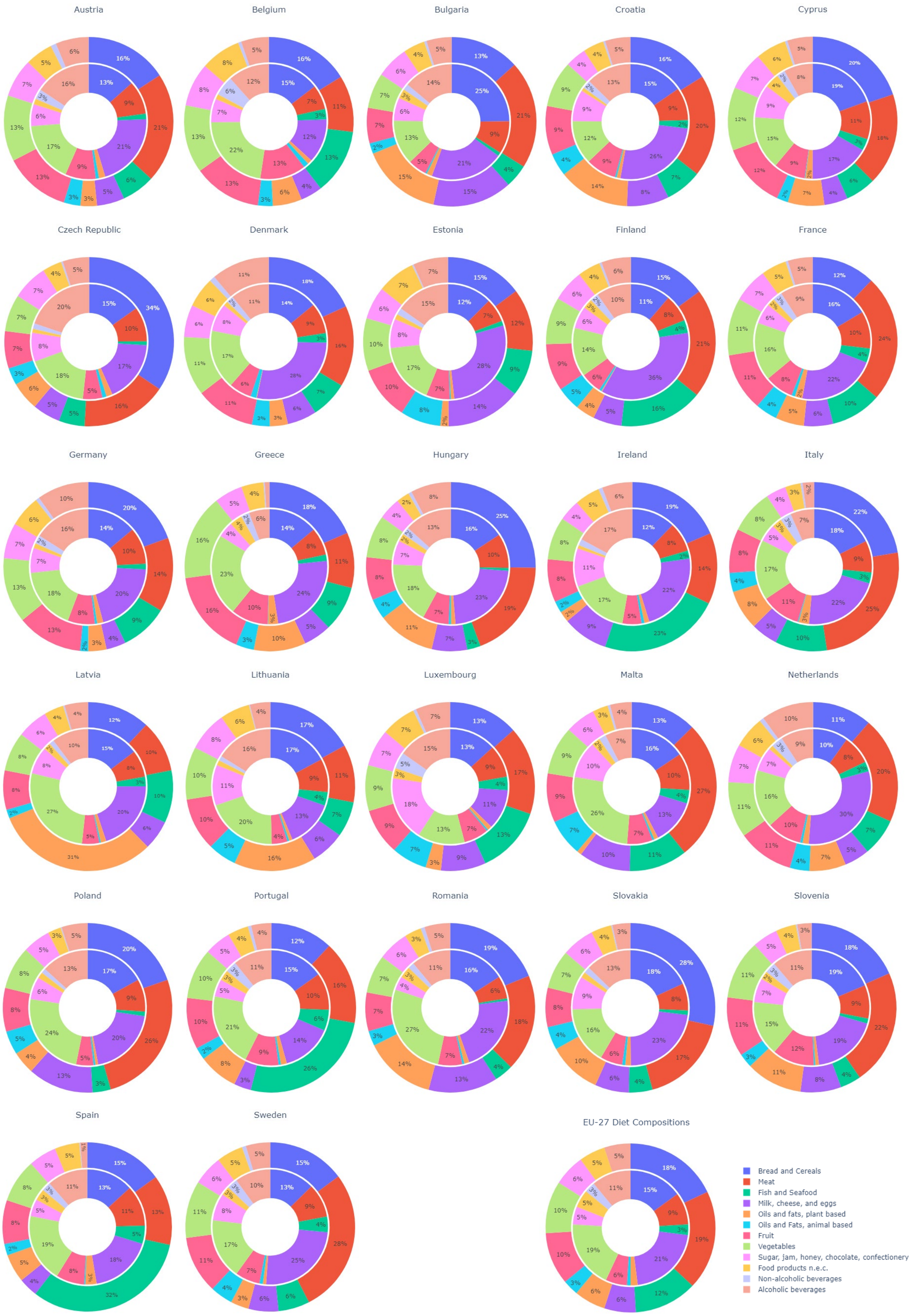

Figure G. Food Footprint of EU-27 countries by geographical origin, in year 2004 and 2014.

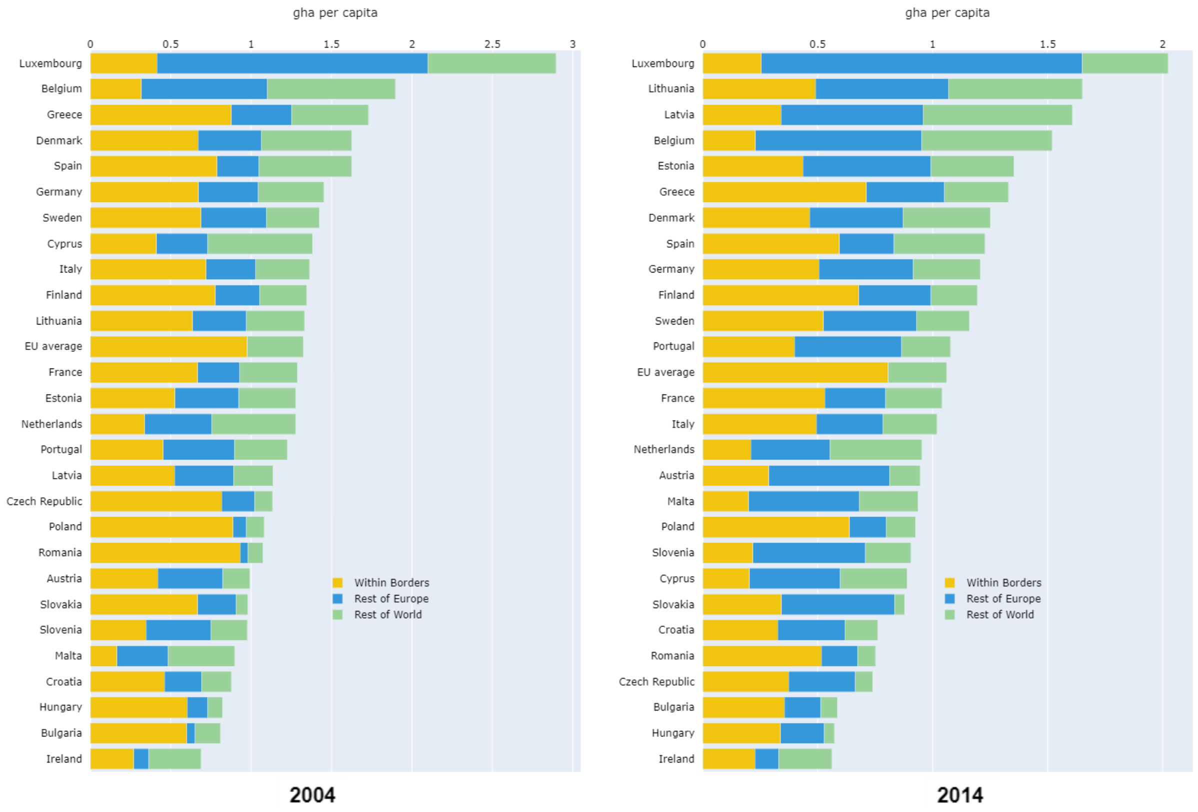

Table 1S. Dietary Footprint intensities and food waste values for EU-27 countries, year 2014. The potential Food Footprint reduction that could be obtained by reducing food waste to zero while mainitaining current Footprint intensities is provided in the last column.

**Year 2014**	**Quantities**	**Calories**	**Food Ecological Footprint**	**Food EF Intensity**	**Food Waste (Total)**	**Food Waste (Household)**	**Food Waste (Food Service)**	**Food Waste (Retail)**
Country	[kg cap^{-1} yr^{-1}]	[kcal cap^{-1} day^{-1}]	[gha cap^{-1} yr^{-1}]	[gm^2 $1000kcal^{-1}$]	[kg cap^{-1} yr^{-1}]	[kg cap^{-1} yr^{-1}]	[kg cap^{-1} yr^{-1}]	[kg cap^{-1} yr^{-1}]
Austria	893	3,667	0.95	7.1	76	39	28	9
Belgium	891	3,787	1.52	11.0	79	50	20	10
Bulgaria	649	2,864	0.59	5.6	112	68	28	16
Croatia	775	2,976	0.76	7.0	123	84	26	13
Cyprus	726	2,993	0.89	8.1	133	95	26	13
Czech Republic	793	3,194	0.74	6.3	108	70	26	13
Denmark	874	3,353	1.25	10.2	132	81	21	30
Estonia	940	3,212	1.35	11.5	99	78	17	5
Finland	983	3,317	1.19	9.9	102	65	23	13
France	828	3,411	1.04	8.4	135	85	24	26
Germany	829	3,540	1.21	9.3	102	75	21	6
Greece	991	3,435	1.33	10.6	175	142	26	7
Hungary	731	3,112	0.57	5.0	132	94	26	13
Ireland	965	3,652	0.56	4.2	124	55	56	13
Italy	903	3,488	1.02	8.0	96	67	26	4
Latvia	858	3,206	1.61	13.7	115	76	26	13
Lithuania	862	3,391	1.65	13.3	115	76	26	13
Luxembourg	929	3,494	2.02	15.9	117	89	21	7
Malta	870	3,414	0.94	7.5	167	129	26	13
Netherlands	882	3,209	0.95	8.1	87	50	26	11
Poland	870	3,412	0.93	7.4	94	56	26	13
Portugal	909	3,377	1.08	8.7	123	84	26	13
Romania	1019	3,415	0.75	6.0	108	70	26	13
Slovakia	668	2,826	0.88	8.5	108	70	26	13
Slovenia	805	3,207	0.91	7.7	61	34	20	7
Spain	866	3,163	1.23	10.6	116	77	26	13
Sweden	799	3,193	1.16	9.9	112	81	21	10
EU-27 (median)	870	3,353	1.02	8.4	115	76.2	25.6	12.8
EU-27 (avg.)	856	3,382	1.06	8.6	113	75.6	25.3	12.0

Figure H. EU-27 Food Footprint (FF) by food macro-categories (right column), appropriated ecological assets (central), and food origin (left) in 2004.

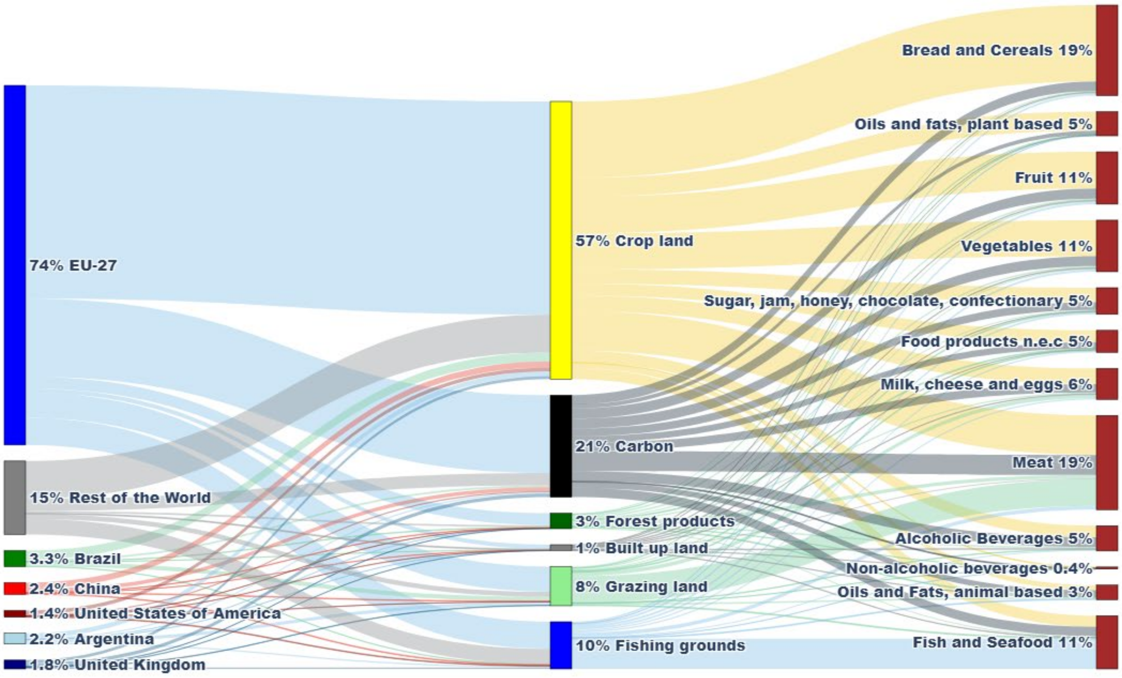

Figure I. EU-27 Food Footprint: countries' reliance on biocapacity outside national borders in 2004 and 2014 – focus on central quadrants with EF ranging between 0.5 and 1.5 gha per person, and external dependency ranging between 40% and 80%. Arrows are used to connect country's positions in the two years; for arrows originating from, or going outside the zoomed boxes please refer to Figure 6 in the main text.

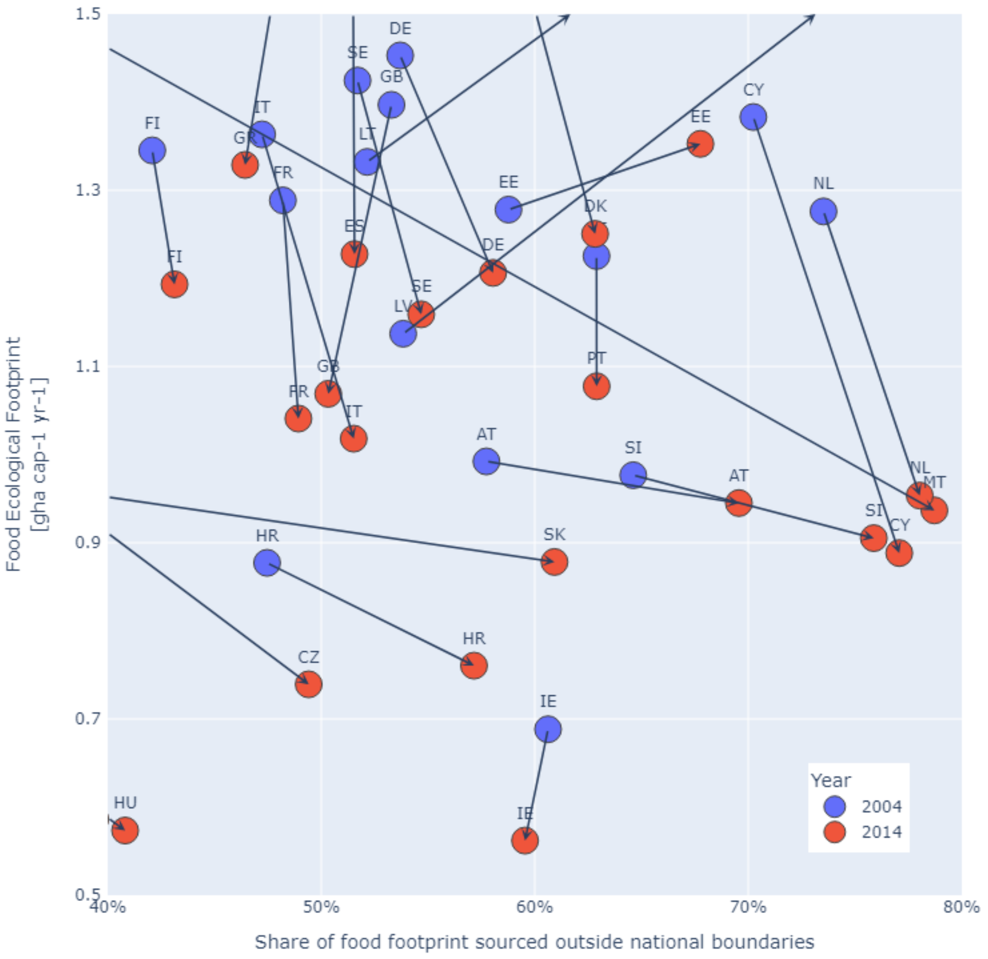

Table 2S. Input data for the calculation of the Ecological Footprint of national production activities. Source: adapted from Borucke et al. (2013).

Dataset	Description	Source
Production of primary agricultural products	Physical quantities (tons) of primary products produced in each of the considered countries	FAO Food and agriculture data, Crop and livestock products. Available at: https://www.fao.org/faostat/en/#data/QCL
Production of crop-based feeds used to feed animals	Data on physical quantities (tons) of feeds, by type of crops, available to feed livestock	Data on Feed from general marketed crops is drawn from FAO Food and agriculture data, Supply Utilization Accounts (2010-), available at: https://www.fao.org/faostat/en/#data/SCL Data on crops grown specifically for fodder is drawn from FAO Food and agriculture data, Crop and livestock products. Available at: https://www.fao.org/faostat/en/#data/QCL
Production of seeds	Data on physical quantities (tons) of seed	FAO Food and agriculture data, Crop and livestock products. Available at : https://www.fao.org/faostat/en/#data/QCL
Livestock crop consumption	Data on crop-based feed for livestock (tons of dry matter per year), split into different crop categories	Calculated by Global Footprint Network based upon the following datasets:<br>• FAO Food and agriculture data, Crop and livestock products. Available at : https://www.fao.org/faostat/en/#data/QCL<br>• Haberl et al. (2007)
Production of primary forest products	Data on physical quantities (tons and m3) of products (timber and wood fuel) produced by each country	FAO Food and agriculture data, Forestry Production and Trade. Available at: https://www.fao.org/faostat/en/#data/FO
Production of primary fishery products	Data on physical quantities (tons) of marine and inland fish and seafood species landed by each country	FAO FishSTAT, Global capture production data. Available at: https://www.fao.org/fishery/statistics-query/en/capture
Carbon dioxide emissions by sector	Data on total amounts of CO_2 emitted by each sector of a country's economy	IEA (International Energy Agency), Greenhouse Gas Emissions from Energy. Available at: https://www.iea.org/data-and-statistics/data-product/greenhouse-gas-emissions-from-energy
Built-up/infrastructure areas	Built-up areas by infrastructure type and country. Except for data drawn from CORINE for EU countries, all other data sources only provide total area values	A combination of data sources is used, in the following order:<br>1. CORINE Land Cover. Available at: https://land.copernicus.eu/pan-european/corine-land-cover<br>2. FAO Food and agriculture data, Land Use. Available at: https://www.fao.org/faostat/en/#data/RL<br>3. Global Agro-Ecological Zones (GAEZ) Model. Available at: https://gaez.fao.org/

		4. Global Land Cover (GLC) 2000. Available at: https://forobs.jrc.ec.europa.eu/glc2000<br>5. Global Land Use Database from the Center for Sustainability and the Global Environment (SAGE) at University of Wisconsin. Available at: https://sage.nelson.wisc.edu/data-and-models/datasets/global-land-use-database/
Cropland yields	World average yield for 164 primary crop products	FAO Food and agriculture data, Crop and livestock products. Available at: https://www.fao.org/faostat/en/#data/QCL
Grazing land yields	World average yield for grass production. It represents the average above-ground edible net primary production for grassland available for consumption by ruminants	Ramankutty, et al., 2008<br>Monfreda et al., 2008
Fish yields	World-average yields for fish species. They are based on the annual marine primary production equivalent	Calculated by Global Footprint Network based on several data sources including:<br>• Sustainable catch value (Gulland, 1971)<br>• Trophic levels of fish species (Fishbase database. Available at www.fishbase.org)<br>• Data on discard factors, efficiency transfer, and carbon content of fish per ton wet weight (Pauly and Christensen, 1995)
Forest yields	World average forest yield. It is based on the forests' Net Annual Increment of biomass.<br>NAI is defined as the average annual volume over a given reference period of gross increment less that of neutral losses on all trees to a minimum diameter of 0 cm (d.b.h.)	World average forest yield calculated by Global Footprint Network based on national Net Annual Increment (NAI) of biomass. NAI data is drawn from two sources:<br>• Temperate and Boreal Forest Resource Assessment – TBFRA (UNECE and FAO, 2000)<br>• Global Fiber Supply Model – GFSM (FAO, 1998)
Average Forest Carbon Sequestration	the capacity of a hectare of world-average forest ecosystem (in t C ha^{-1} yr^{-1}) to sequester atmospheric carbon dioxide through photosynthesis.	Mancini et al., 2016

Table 3S. GTAP 10 sectors' list.

Number	Code	Detailed sector description
1	pdr	Paddy rice
2	wht	Wheat
3	gro	Cereal grains nec
4	v_f	Vegetables, fruit, nuts
5	osd	Oil seeds
6	c_b	Sugar cane, sugar beet
7	pfb	Plant-based fibers
8	ocr	Crops nec
9	ctl	Bovine cattle, sheep and goats, horses
10	oap	Animal products nec
11	rmk	Raw milk
12	wol	Wool, silk-worm cocoons
13	frs	Forestry
14	fsh	Fishing
15	coa	Coal
16	oil	Oil
17	gas	Gas
18	oxt	Other Extraction (formerly omn Minerals nec)
19	cmt	Bovine meat products
20	omt	Meat products nec
21	vol	Vegetable oils and fats
22	mil	Dairy products
23	pcr	Processed rice
24	sgr	Sugar
25	ofd	Food products nec
26	b_t	Beverages and tobacco products
27	tex	Textiles
28	wap	Wearing apparel
29	lea	Leather products
30	lum	Wood products
31	ppp	Paper products, publishing
32	p_c	Petroleum, coal products
33	chm	Chemical products
34	bph	Basic pharmaceutical products
35	rpp	Rubber and plastic products
36	nmm	Mineral products nec
37	i_s	Ferrous metals
38	nfm	Metals nec
39	fmp	Metal products
40	ele	Computer, electronic and optical products
41	eeq	Electrical equipment
42	ome	Machinery and equipment nec
43	mvh	Motor vehicles and parts
44	otn	Transport equipment nec
45	omf	Manufactures nec

46	ely	Electricity
47	gdt	Gas manufacture, distribution
48	wtr	Water
49	cns	Construction
50	trd	Trade
51	afs	Accommodation, Food and service activities
52	otp	Transport nec
53	wtp	Water transport
54	atp	Air transport
55	whs	Warehousing and support activities
56	cmn	Communication
57	ofi	Financial services nec
58	ins	Insurance (formerly isr)
59	rsa	Real estate activities
60	obs	Business services nec
61	ros	Recreational and other services
62	osg	Public Administration and defense
63	edu	Education
64	hht	Human health and social work activities
65	dwe	Dwellings

Figure J. FBS macro-categories that were split in sub-categories through the CPC classification to match with proper COICOP categories. The % of allocation of each sub-category is also shown.

FBS code	Food Balance Sheet (FBS) category	CPC code	CPC 2.1 category	COICOP category	Allocation factor
2555	Soyabeans and products	141	Soybeans	Vegetables	99.7%
		2399	Soya sauce	Product nec	0.3%
2558	Rape and Mustardseed and products	1442	Mustard seed	Fruit	99%
		2399	Flour of mustard seed	Product nec	1%
2601	Tomatoes and products	1234	tomatoes	Vegetables	61%
		2132	tomato juice	Non-alcoholic beverage	0.1%
		2139	paste of tomatoes	Product nec	38%
		2139	tomatoes, peeled (o/t vinegar)		
2605	Vegetables, Other and products		All vegetable	Vegetables	92%
		1221	Watermelons	Fruit	0.7%
		2399	homogenized vegetable preparations	Product nec	0.1%
2611	Oranges, Mandarines and products		Oranges, tangerines, mandarins, clementines	Fruit	66%
		2143	orange juice	Non-alcoholic	34%
			orange juice, concentrated		
			juice of tangerine		
2612	Lemons, Limes and products	1322	lemons and limes	Fruit	93%
		2143	juice of lemon	Non-alcoholic	7%
			Lemon juice, concentrated		
2613	Grapefruit and products	1321	pomelos and grapefruits	Fruit	79%
		2143	grapefruit juice	Non-alcoholic	21%
		2143	grapefruit juice, concentrated		
2614	Citrus, Other	1329	other citrus fruit, n.e.c.	Fruit	0%
		2143	Juice of citrus fruit n.e.c.	Non-alcoholic	100%
		2143	Citrus juice, concentrated n.e.c.		
2617	Apples and products	1341	apples	Fruit	71%
		2143	apple juice	Non-alcoholic	29%
			apple juice, concentrated		
2618	Pineapples and products	1318	pineapples	Fruit	85%
		2143	pineapple juice	Non-alcoholic	15%
		2149	pineapples, otherwise prepared or preserved		
		2143	juice of pineapples, concentrated		
2620	Grapes and products (excl wine)	1330	grapes	Fruit	87%
		2141	raisins		
		2421	must of grape		
		2143	grape juice	Non-alcoholic	13%
2625	Fruits, other		Various type of fruits	Fruits	58%
		2143	Juice of plum	Non-alcoholic beverages	11%
		2143	Juice of plum, concentrated		
		2143	Juice of mango		
		2143	juice of fruits n.e.c.		
		2399	homogenized cooked fruit, prepared	Food product nec	31%
		F062	fruit prepared n.e.c.		
2635	Tea (including mate) and products	1620	tea leaves	Food product nec	89%
		1630	maté leaves	Non-alcoholic beverages	11%
		2391	extracts, essences and concentrates of tea or mate, and preparations with a basis thereof or with a basis of tea or maté		
2848	Milk - Excluding Butter		All milk products	Milk, cheese, and eggs	94%
		2227	ice cream and other edible ice [only calories]	Sugar, jam, honey, chocolate, confectionery	6%

References (cited in Tables)

Borucke, M., Moore, D., Cranston, G., Gracey, K., Iha, K., et al., (2013). Accounting for demand and supply of the Biosphere's regenerative capacity: the National Footprint Accounts' underlying methodology and framework. Ecological Indicators 24, 518–533.

FAO, (1998). Global Fiber Supply Model. ftp://ftp.fao.org/docrep/fao/006/X0105E/X0105E.pdf (accessed February 2011).

Gulland, J.A. (1971). The Fish Resources of the Ocean. Fishing News, West Byfleet, Surrey, United Kingdom

Haberl, H., Erb, K.H., Krausmann, F., Gaube, V., Bondeau, A., Plutzar, C., Gingrich, S., Lucht, W., Fischer-Kowalski, M. (2007). Quantifying and mapping the human appropriation of net primary production in earth's terrestrial ecosystems. Proc. Natl. Acad. Sci. U.S.A. 104, 12942–12947.

Mancini, M.S., Galli, A., Niccolucci, V., Lin, D., Bastianoni, S., Wackernagel, M., Marchettini, N. (2016). Ecological Footprint: Refining the carbon Footprint calculation. Ecol. Ind. 61, 390–403.

Monfreda, C., Ramankutty, N., Foley, J.A. (2008). Farming the planet: 2. Geographic distribution of crop areas, yields, physiological types, and net primary production in the year 2000. Glob. Biogeochem. Cycles 22, GB1022, https://doi.org/10.1029/2007GB002947.

Pauly, D., Christensen, V. (1995). Primary production required to sustain global fisheries. Nature 374, 255–257.

Ramankutty, N., Evan, A. T., Monfreda, C. & Foley, J.A. (2008). Farming the planet: 1. Geographic distribution of global agricultural lands in the year 2000. Glob. Biogeochem. Cycles 22, GB1003, https://doi.org/10.1029/2007GB002952.

UNECE and FAO (2000). Temperate and Boreal Forest Resource Assessment. UNECE, FAO, Geneva.

References (further readings)

Banach, J.L., van der Berg, J.P., Kleter, G., van Bokhorst-van de Veen, H., Bastiaan-Net, S., Pouvreau, L., & van Asselt, E.D. Alternative proteins for meat and dairy replacers: Food safety and future trends. *Crit Rev Food Sci Nutr*. **27**:1-18 (2022) https://doi.org/10.1080/10408398.2022.2089625

Batlle-Bayer, L., Bala, A., García-Herrero, I., Lemaire, E., Song, G., Aldaco, R. et al. The Spanish Dietary Guidelines: A potential tool to reduce greenhouse gas emission of current dietary patterns. *J Clean Prod*, **213**, 588-98 (2019). DOI 10.1016/j.jclepro.2018.12.215

Blackstone, N.T., El-Abbadi, N., McCabe, M.S., Griffin, T., & Nelson, M. Linking sustainability to the healthy eating patterns of the Dietary Guidelines for Americans: a modelling study. *The Lancet Planetary Health*, **2**(8): e344-e352 (2018).

Bruckner, M., Häyhä, T., Giljum, S., Maus, V., Fischer, G., Tramberend, S., & Börner, J. Quantifying the global cropland footprint of the European Union's non-food bioeconomy. *Environ. Res. Lett*., **14**, 045011 (2019a).

Bruckner, M. Wood, R., Moran, D., Kuschnig, N., Wieland, H., Maus, V., & Börner, J. FABIO—The Construction of the Food and Agriculture Biomass Input–Output Model. *Environmental Science and Technology,* **53**, 11302–11312 (2019b).

Bruno, M., Thomsen, M., Pulselli, F.M., Patrizi, N., Marini, M., & Caro, D. The carbon footprint of Danish diets. *Climatic Change*, **156**(4), 489–507 (2019).

Caro, D. Sustainability of food systems and reinforcement of the science-policy interface: Re-focusing on priorities. *Resources, Environment and Sustainability*, **11**, 100100 (2023).

Chatellier, V. Review: International trade in animal products and the place of the European Union: main trends over the last 20 years. *Animal*, **15**(1), 100289 (2021). https://doi.org/10.1016/j.animal.2021.100289

Fanzo, J. Achieving equitable diets for all: the long and winding road. *One Earth*, **4**(4), 470-473, (2021). https://doi.org/10.1016/j.oneear.2021.03.007

Fardet, A., Rock, E. Ultra-Processed Foods and Food System Sustainability: What Are the Links? *Sustainability*, **12**(15):6280 (2020).

Galli, A., Wackernagel, M., Iha, K., Lazarus, E. Ecological footprint: implications for biodiversity. *Biol. Conserv*. **173**, 121–132 (2014).

Gibbs, J., Cappuccio, F.P. Plant-Based Dietary Patterns for Human and Planetary Health. *Nutrients*, **14**, 1614 (2022).

Halpern, B.S., Frazier, M., Verstaen, J., et al. The environmental footprint of global food production. *Nature Sustainability*, **5**, 1027–1039 (2021). https://doi.org/10.1038/s41893-022-00965-x.

Herrero, M., Henderson, B., Havlík, P., et al. Greenhouse gas mitigation potentials in the livestock sector. *Nat. Clim. Change*, **6**, 452–461 (2016).

Herrero, M., Thornton, P.K., Mason-D'Croz, D., et al. Innovation can accelerate the transition towards a sustainable food system. *Nat. Food*, **1**, 266-272, (2020). 10.1038/s43016-020-0074-1

Ivanova, D., & Wood, R. The unequal distribution of household carbon footprints in Europe and its link to sustainability. *Global Sustainability*, **3**, E18 (2020). doi:10.1017/sus.2020.12

Ivanova, D., Stadler, K., Steen-Olsen, K., et al. Environmental impact assessment of household consumption. *Journal of Industrial Ecology*, **20**, 526–536. (2015). doi: 10.1111/jiec.12371

Kitzes, J. An Introduction to Environmentally-Extended Input-Output Analysis. *Resources*, **2**, 489-503. (2013). https://doi.org/10.3390/resources2040489

Kitzes, J., Galli, A., Bagliani, M., Barrett, J., Dige, G., Ede, S., Erb, K., Giljum, S., Haberl, H., Hails, C., Jungwirth, S., Lenzen, M., Lewis, K., Loh, J., Marchettini, N., Messinger, H., Milne, K., Moles, R., Monfreda, C., et al. A research agenda for improving national Ecological Footprint accounts. *Ecological Economics*, **68**, 1991–2007 (2009).

Kusaka, S., Ishimaru, E., Hyodo, F. et al. Homogeneous diet of contemporary Japanese inferred from stable isotope ratios of hair. *Sci Rep* **6**, 33122 (2016). https://doi.org/10.1038/srep33122

Lenzen, M., Moran, D., Kanemoto, K. & Geschke, A. Building eora: a global multi-region input–output database at high country and sector resolution. *Economic Systems Research*, **25**, 20–49 (2013).

Mertens, E., Kuijsten, A., van Zanten, H.H.E., et al. Dietary choices and environmental impact in four European countries. *Journal of Cleaner Production*, **237**, 117827 (2019). https://doi.org/10.1016/j.jclepro.2019.117827

Monteiro, C.A., Moubarac, J.-C., Bertazzi Levy, R., et al. Household availability of ultra-processed foods and obesity in nineteen European countries. *Public Health Nutr*. **21**(1), 18-26 (2018). doi: 10.1017/S1368980017001379.

Moran, D., Wood, R., Hertwich, E., et al. Quantifying the potential for consumer-oriented policy to reduce European and foreign carbon emissions. *Climate Policy*, **20**, :sup1, S28-S38 (2020). https://doi.org/10.1080/14693062.2018.1551186

Osei-Owusu, A.K., Wood, R., Bjelle, E.L., Caro, D., & Thomsen, M. Understanding the trends in Denmark's global food trade-related greenhouse gas and resource footprint. *Journal of Cleaner Production*, **313**, 127785 (2021).

Popkin, B. Ultra-processed foods' impacts on health. 2030 – Food, Agriculture and rural development in Latin America and the Caribbean, No. 34. Santiago de Chile. FAO. (2020).

Sala S., Sanyé Mengual E. Consumption Footprint: Assessing the Environmental Impacts of EU Consumption, European Commission. JRC126257 (2022). Available at : https://publications.jrc.ec.europa.eu/repository/handle/JRC126257

Springmann, M., Godfray, H.C.J., Rayner, M., Scarborough, P. et al. Analysis and valuation of the health and climate change co-benefits of dietary change. *PNAS*, **113**(15), 4146-4151 (2016). https://doi.org/10.1073/pnas.1523119113

Stadler, K., Wood, R., Bulavskaya, T., et al. EXIOBASE 3: Developing a Time Series of Detailed Environmentally Extended Multi-Regional Input-Output Tables. *Journal of Industrial Ecology*, **22**, 502–515 (2018).

Steen-Olsen, K., Weinzettel, J., Cranston, G., Ercin, A. E., & Hertwich, E.G. Carbon, Land, and Water Footprint Accounts for the European Union: Consumption, Production, and Displacements through International Trade. *Environmental Science and Technology*, **46**(20), 10883–10891 (2012).

Steffen, W., Richardson, K., Rockström, J., Cornell, S.E., Fetzer, I., Bennett, E.M., & De Wit, C.A. Planetary boundaries: guiding human development on a changing planet. *Science*, **347**(6223), 1259855 (2015).

Tsuchiya, K., Iha, K., Murthy, A., Lin, D., Altiok, S., Rupprecht, C.D.D., Kiyono, H., & McGreevy, S.R. Decentralization and local food: Japan's regional Ecological Footprints indicate localized sustainability strategies. *Journal of Cleaner Production*, **292**, 126043 (2021). https://doi.org/10.1016/j.jclepro.2021.126043

Vanham, D., Medarac, H., Schyns, J.F. et al. The consumptive water footprint of the European Union energy sector. *Environ. Res. Lett.*, **14**, 104016 (2019). DOI 10.1088/1748-9326/ab374a.
