# Supplementary material for: EU-27 ecological footprint was primarily driven by food consumption and exceeded regional biocapacity from 2004 to 2014
Source: Nat Food. 2023 Sep 14;4(9):810–22. doi: 10.1038/s43016-023-00843-5 (PMC10513931; doi:10.1038/s43016-023-00843-5)
Supplement: Supplementary file 2 — Reporting Summary [file 43016_2023_843_MOESM2_ESM.pdf]

nature portfolio

Corresponding author(s): Alessandro Galli

Last updated by author(s): Aug 8, 2023

Reporting Summary

Nature Portfolio wishes to improve the reproducibility of the work that we publish. This form provides structure for consistency and transparency in reporting. For further information on Nature Portfolio policies, see our Editorial Policies and the Editorial Policy Checklist.

Statistics

For all statistical analyses, confirm that the following items are present in the figure legend, table legend, main text, or Methods section.

n/a	Confirmed	
☒	☐	The exact sample size (*n*) for each experimental group/condition, given as a discrete number and unit of measurement
☒	☐	A statement on whether measurements were taken from distinct samples or whether the same sample was measured repeatedly
☒	☐	The statistical test(s) used AND whether they are one- or two-sided<br>*Only common tests should be described solely by name; describe more complex techniques in the Methods section.*
☒	☐	A description of all covariates tested
☒	☐	A description of any assumptions or corrections, such as tests of normality and adjustment for multiple comparisons
☒	☐	A full description of the statistical parameters including central tendency (e.g. means) or other basic estimates (e.g. regression coefficient) AND variation (e.g. standard deviation) or associated estimates of uncertainty (e.g. confidence intervals)
☒	☐	For null hypothesis testing, the test statistic (e.g. *F*, *t*, *r*) with confidence intervals, effect sizes, degrees of freedom and *P* value noted<br>*Give P values as exact values whenever suitable.*
☒	☐	For Bayesian analysis, information on the choice of priors and Markov chain Monte Carlo settings
☒	☐	For hierarchical and complex designs, identification of the appropriate level for tests and full reporting of outcomes
☒	☐	Estimates of effect sizes (e.g. Cohen's *d*, Pearson's *r*), indicating how they were calculated

Our web collection on statistics for biologists contains articles on many of the points above.

Software and code

Policy information about availability of computer code

Data collection	No software was used to collect data
Data analysis	Data was analyzed using Matlab version R2014a x64 and mySQL version 8.0.27

For manuscripts utilizing custom algorithms or software that are central to the research but not yet described in published literature, software must be made available to editors and reviewers. We strongly encourage code deposition in a community repository (e.g. GitHub). See the Nature Portfolio guidelines for submitting code & software for further information.

Data

Policy information about availability of data

All manuscripts must include a data availability statement. This statement should provide the following information, where applicable:

- Accession codes, unique identifiers, or web links for publicly available datasets
- A description of any restrictions on data availability
- For clinical datasets or third party data, please ensure that the statement adheres to our policy

Data supporting the findings of this study is available from multiple sources (e.g., FAO, IEA, GTAP) as described in the methodology section (see Table 2S in the Supplementary Material for further details on each individual dataset and how to access it). Some of this data is freely available (and provided as raw data in the Excel file 'EU-27 Food Footprint_source data.xlsx') while restrictions apply to the availability of other data (i.e., GTAP version 10), which was used under license for

the current study, and is thus not publicly available. Data other than that included in the ‘EU-27 Food Footprint_source data.xlsx’ file is available from the corresponding author upon request. Visualization of aggregated results is publicly available at: https://food.footprintnetwork.org/

Human research participants

Policy information about studies involving human research participants and Sex and Gender in Research.

Reporting on sex and gender	No human participant was involved in this study
Population characteristics	*Describe the covariate-relevant population characteristics of the human research participants (e.g. age, genotypic information, past and current diagnosis and treatment categories). If you filled out the behavioural & social sciences study design questions and have nothing to add here, write "See above."*
Recruitment	*Describe how participants were recruited. Outline any potential self-selection bias or other biases that may be present and how these are likely to impact results.*
Ethics oversight	*Identify the organization(s) that approved the study protocol.*

Note that full information on the approval of the study protocol must also be provided in the manuscript.

Field-specific reporting

Please select the one below that is the best fit for your research. If you are not sure, read the appropriate sections before making your selection.

☐ Life sciences ☐ Behavioural & social sciences ☒ Ecological, evolutionary & environmental sciences

For a reference copy of the document with all sections, see nature.com/documents/nr-reporting-summary-flat.pdf

Ecological, evolutionary & environmental sciences study design

All studies must disclose on these points even when the disclosure is negative.

Study description	The study uses a known environmental accounting methodology (i.e., Ecological Footprint) to assess the environmental impacts associated with food systems in the EU-27 and Member States.
Research sample	The analysis is conducted for the EU-27 region and each individual Member State, and the residents therein.
Sampling strategy	No method was used to determine sample size as the Footprint assessment was conducted by means of national statistical data drawn from official international databases.
Data collection	Input data (e.g., from FAO, IEA, GTAP) is downloaded by the researchers involved in this study from international datasets. All collected/downloaded data is then stored in Global Footprint Network's internal database (my SQL).
Timing and spatial scale	Most input data (e.g., from FAO, IEA) is downloaded annually by the researchers involved in this study; GTAP data, however, is downloaded every time a new edition of such dataset is released (usually every 4-5 years).
Data exclusions	No data was excluded from the analysis.
Reproducibility	All attempts to repeat the analysis (conducted by different researchers) were successful.
Randomization	Randomization is not relevant for this study as there was no need to eliminate sample selection biases.
Blinding	Blinding is not relevant for this study.

Did the study involve field work? ☐ Yes ☒ No

Reporting for specific materials, systems and methods

We require information from authors about some types of materials, experimental systems and methods used in many studies. Here, indicate whether each material, system or method listed is relevant to your study. If you are not sure if a list item applies to your research, read the appropriate section before selecting a response.

Materials & experimental systems

n/a	Involved in the study
☒	☐ Antibodies
☒	☐ Eukaryotic cell lines
☒	☐ Palaeontology and archaeology
☒	☐ Animals and other organisms
☒	☐ Clinical data
☒	☐ Dual use research of concern

Methods

n/a	Involved in the study
☒	☐ ChIP-seq
☒	☐ Flow cytometry
☒	☐ MRI-based neuroimaging
